# Activity and Enantioselectivity of Hydroxynitrile Lyase from *Granulicella tundricula:* Computational Insights

**DOI:** 10.1021/acs.inorgchem.6c02524

**Published:** 2026-08-22

**Authors:** Mario Prejanò, Ulf Hanefeld, Tiziana Marino

**Affiliations:** † Department of Chemistry and Chemical Technologies, University of Calabria, Via Pietro Bucci 14/C, 87036 Arcavacata di Rende, CS, Italy; ‡ Biokatalyse, Afdeling Biotechnologie, 2860 Technische Universiteit Delft, Van der Maasweg 9, 2629 HZ Delft, The Netherlands

## Abstract

Hydroxynitrile lyase from *Granulicella tundricola* (*Gt*HNL) is a Mn­(II)-dependent enzyme that catalyzes the *R*-selective synthesis of cyanohydrins. Mn­(II) is pentacoordinated by the enzyme in a distorted octahedral 17 electron complex. The aldehyde coordinates to the vacant site of Mn­(II), and together with H106 as a base, the Lewis acid catalyzes the enantioselective synthesis. This study performed in the framework of density functional theory aims to elucidate the reaction mechanism examining the source of enantioselectivity of *Gt*HNL in the reaction between benzaldehyde and HCN to obtain *R*-mandelonitrile. Starting from a model built from the available crystallographic structure with its distorted octahedral Mn­(II), different binding modes of the substrates are first evaluated, followed by the calculation of the mechanistic pathway (transition states and intermediates) from each complex obtained. The effect of amino acid mutations at the active site on the enzyme reactivity was further discussed. The calculated energies are consistent with experimental observations, and the analysis of transition-state geometries provides insights into the origins of enantioselectivity of the enzyme.

## Graphical abstract

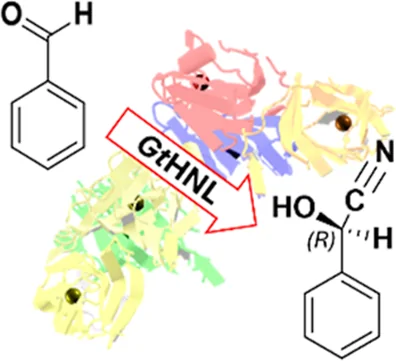

## Introduction

The carbon–carbon bond is central to organic chemistry. Its enzymatic formation was the first enantioselective synthesis ever described.^1^ The reaction was catalyzed by the hydroxynitrile lyase (HNL) from *Prunus amygdalus* (almonds, *Pa*HNL), and the reaction has attracted attention ever since (Scheme 1A).^2–4^ After a flurry of research a hundred years ago, a range of enzymes were known, and the topic was revitalized once enantioselectivity attracted attention again in the 1960s. After more than one hundred years of research, many HNLs are known. Their convergent evolution in several kingdoms of life but mostly in plants has led to a wide range of different protein scaffolds forming the backbone of this chemistry, catalyzing the reaction with many different mechanisms.^4,5^ Remarkably, the vast majority of the HNLs are *R*-selective, irrespective of their mechanism or the protein scaffold.^2,3,6^ Only three are known to be *S*-selective, the closely related HNLs from *Hevea brasiliensis* (*Hb*HNL), *Baliospermum montanum* (*Bm*HNL), and *Manihot esculenta* (*Me*HNL), all evolved in the α/β-hydrolase fold. A fourth member of these α/β-hydrolase fold HNLs is *R*-selective, the HNL from *Arabidopsis thaliana* (*At*HNL).^7^ The enantioselectivity is thus not dependent on the structure and initial enzyme activity from which the HNL activity evolved. Structural, biochemical, and computational studies have convincingly clarified the mechanism and the different ways of stereoinduction for these α/β-hydrolase fold enzymes.^7–12^

**Scheme 1:**
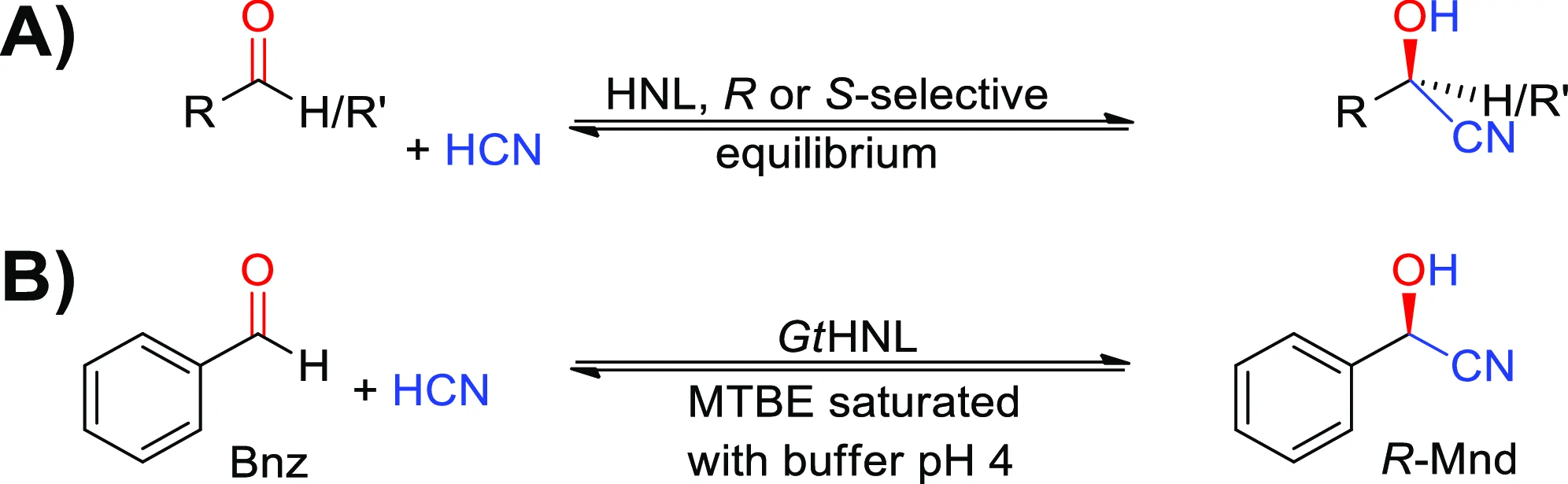
(A) HNLs in Nature Catalyze the Degradation of Cyanohydrins and Can Be Used in Laboratories and Industries to Synthesize Enantiopure Cyanohydrins; (B) *Gt*HNL Catalyzes the Formation of *R*-Mandelonitrile (*R*-**Mnd**) from Benzaldehyde (**Bnz**) and **HCN** ^a^

a The reactions can be performed in aqueous biphasic mixtures or in a buffer-saturated organic solvent.

For the more recently described *R*-selective HNL from *Granulicella tundricola* (*Gt*HNL; Scheme 1B), much less information is available.^13–16^

The crystal structure of this cupin has been elucidated for both the wild type and mutants of *Gt*HNL.^13,14,16^ The HNL activity was initially not expected for this and other cupins as they were isolated from microbial sources rather than plants, in which most HNLs were found. Biochemical studies of this and other cupin-based HNLs in general demonstrated the importance of the metal and its coordination in the active site.^17^ The pentacoordinated high-spin Mn­(II) center is crucial to the catalytic activity of *Gt*HNL.^18^ Four histidines and one amide (H53, H55, Q59, H94, and H96) coordinate the divalent metal in an octahedral 17 electron complex, the only accessible site of the metal being occupied by water. EPR studies revealed the importance of the distorted character of the Mn­(II) complex, activating the metal to be sufficiently Lewis acidic to catalyze the reaction via the pentacoordinated state. This is in contrast to the chemical Mn-based catalysts; they only provide high enantioselectivity and activity with Mn­(III).^19,20^ Moreover, EPR clarified the mode of action of these Mn­(II), coordinating the carbonyl compound and activating it, while the HCN is deprotonated by a histidine residue (H106). A hydrogen-bonding network involving also H96 might aid this deprotonation and even help to stabilize the developing oxyanion (see Scheme 2).^18^ These findings raise several questions. How does the distorted structure of the Mn­(II) enhance Lewis acidity? How are the charges distributed during catalysis to enable deprotonation? And above all, how is the enantioselectivity induced? Understanding these parameters would help to evolve the cupin-type HNLs into even more active catalysts with the possibility to induce *S*-selective formation of cyanohydrins. This would, in turn, enable an even wider application of these powerful catalysts in synthesis, both in the laboratory^4^ and in industry.^21^

**Scheme 2:**
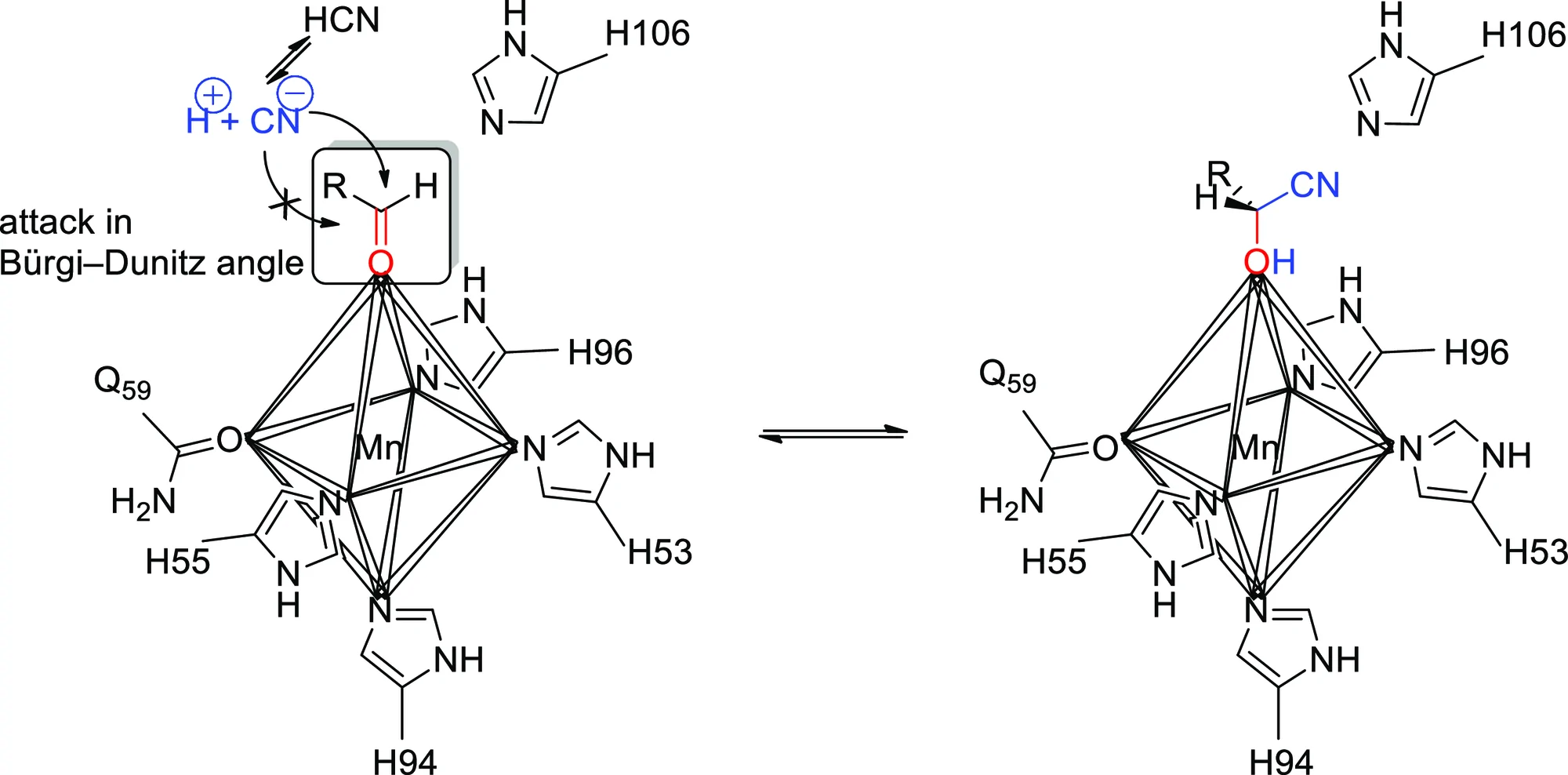
Proposed Mechanism of Action of *Gt*HNL^a^

a Coordination of the carbonyl group to Mn­(II) and subsequent attack of the cyanide ion along the Bürgi–Dunitz angle yields the chiral cyanohydrins. His106 is involved in the deprotonation of HCN.

Moreover, it would aid the further exploration of the promiscuous activities of *Gt*HNL^16,22^ and HNLs in general.^4,23,24^ With these questions in mind, an extensive computational investigation, based on density functional theory (DFT), on the main aspects that rule the activity of *Gt*HNL is reported. An initial model of the metal and its first-coordination sphere is built to study inherent electronic properties of the catalytic center, while the reaction mechanism of *Gt*HNL has been investigated in the framework of quantum chemical cluster approach. This modeling scheme, due to its robustness and versatility, has been largely utilized for disclosing and explaining the reactivity and enantioselectivity of a number of enzymes.^25–29^ For this purpose, an active site model of *Gt*HNL has been designed, starting from a recent characterized crystallographic structure (PDB 4BIF).^13^

The results of the present investigation are in very good agreement with the available experimental data and, in addition, provide additional insights into the origin of the stereospecificity at the molecular level, which are helpful to drive the rational design of biocatalysts with new and tailored reactivity of *Gt*HNL.

## Methods

### Active Site Model

To investigate the reactivity of the *Gt*HNL enzyme, an active-site model was built up based on the X-ray crystallographic structure (PDB 4BIF).^13^ The model includes the Mn­(II) ion along with amino acid residues from both the first-coordination sphere (H53, H55, Q59, H94, and H96) and the second-coordination sphere (F19, V42, T50, H106, A108, Q110, V118, and W120), as illustrated in Figure 1. One crystallographic water molecule (w1) was retained, while benzaldehyde (**Bnz**) and **HCN** were manually positioned within the active site, with the former coordinating Mn­(II) and the latter located in **Bnz** proximity for the occurrence of the reaction (see the next paragraphs for further details). The choice of focusing on the main species in the active site involved in the reaction, i.e., substrates, water, metal cation, and its first- and second-coordination spheres, corresponds to a main approximation of the QM cluster approach in which the rest of the enzyme can be modeled via an implicit solvation model, as discussed in the next sections. Interested readers can find comparison of the QM cluster approach with other modeling schemes, such as hybrid QM/MM, using whole enzymes in the literature.^30^

**Figure 1:**
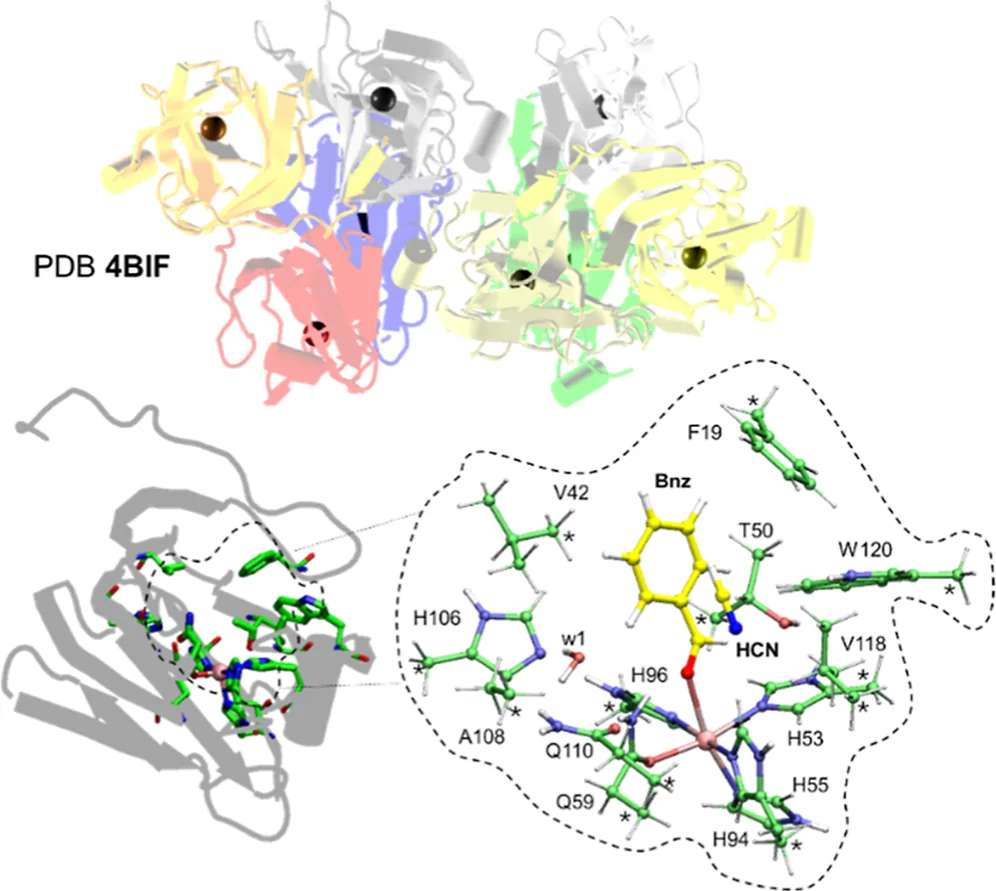
On the left, a cartoon representation of the crystallographic structure of the two-homotetramers of *Gt*HNL protein (PDB 4BIF), with metal species represented as spheres. On the right, the active site model adopted for the study of *Gt*HNL reactivity. Atoms kept fixed during the geometry optimizations are labeled with an asterisk.

After preliminary tests on the model’s size, the current one was intentionally built considering amino acid parts in direct contact with the substrates, to study the inherent activity of the enzyme.

Following the QM-cluster modeling approach, amino acid residues were truncated at peripheral carbon atoms and capped with hydrogen atoms (marked with “*” in Figure 1). These capped carbon atoms were fixed at their initial positions during geometry optimization. This strategy minimizes unrealistic structural distortions of the residues, while still allowing sufficient flexibility for the system to adjust during the reaction, consistent with previous mechanistic investigations.^25–29,31–33^ In addition, this modeling scheme leads to small imaginary frequencies (<50*i* cm^–1^) that do not affect the relative energies, as observed previously.^25–29,31–33^

The final model consists of 187 atoms (12 of them anchored), and the total charge is +2. A smaller model of Mn­(II) and its first-coordination sphere (H53, H55, Q59, H94, and H96) was selected to study the electronic properties of the metal-containing active site. The high spin multiplicity state of Mn was considered in all the calculations (S = 5/2), in agreement with previous experimental observations.^18^ As commonly required in the applied computational protocol, this choice was further validated by optimizing lower spin multiplicities (S = 3/2 and S = half), which yielded higher energies (see Figure S1), where the key geometrical parameters are also reported. The spin densities for all optimized species are summarized in Table S1.

### Technical details

For the current investigation, all the calculations were carried out using the Gaussian 16C.01 program^34^ and the B3LYP-D3­(BJ) functional.^35–38^ In geometry optimizations, the 6-31G­(d,p) basis set was employed for H, C, N, and O atoms, while the SDD pseudopotential and its relative basis set were selected for the treatment of Mn.^39^ At the same level of theory, frequencies were calculated to extrapolate zero-point energy corrections (ZPE), and the effect of the surrounding was estimated via single-point energy calculations, with implicit solvation model SMD,^40^ by considering protein and water-exposed environments in accordance to the dielectric constant values, ε = 4.0 and ε = 78.3, respectively. Starting from enzyme–substrate complexes, minima and maxima were identified performing relaxed energy scan of the reaction coordinates involved in the chemical event, and frequencies calculation was used to confirm their nature of intermediates or transition (imaginary frequency for the first ranked normal mode), respectively. For each intermediate, geometry was further validated by full optimization starting from the linked transition states. The larger 6-311 + G­(2d,2p) basis set, coupled with SDD, was employed for single-point energy calculations on the optimized geometries, to obtain more accurate electronic energies.

The procedure described has been successfully adopted to study other metal-containing systems.^32,33^ The final energies discussed in the paper are thus concerning the large basis set, corrected with solvation and ZPE energies. For comparative analysis purposes with available experimental data, the final energies were assumed to be comparable to Gibbs free energies.

## Results and Discussion

### Mn­(II) Distortion

As introduced above, EPR studies indicated that the catalysis promoted by *Gt*HNL is directly related to the distorted Lewis acidic Mn­(II) complex.^17,18^ However, how this distortion impacts the catalytic activity of the enzyme was not fully understood.

Using a simplified model consisting of the metal, in the pentacoordinated state, and of the amino acid residues of its first-coordination sphere, the effect of the distortion has been investigated, constraining the O_Q_–Mn–N_H3_ angle (see Figure 2A). Therefore, the empty axial coordination site of the metal and the lowest unoccupied molecular orbital (LUMO), characterized by the d* atomic orbital of Mn­(II) required for the binding of the substrate, have been calculated.

**Figure 2:**
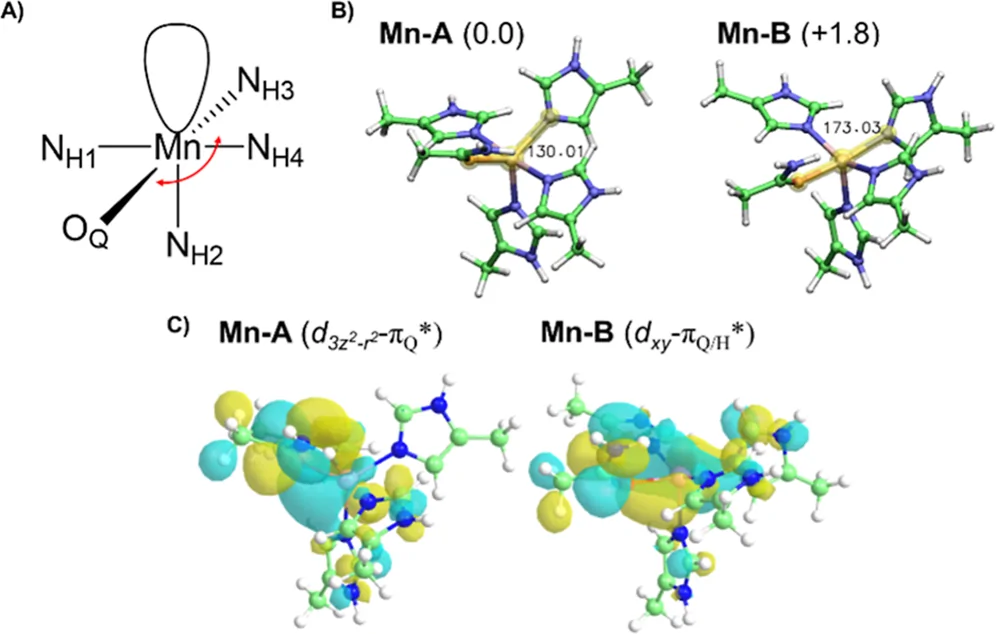
(A) Scheme of the first-coordination sphere of Mn­(II) in *Gt*HNL, (B) optimized structures of **Mn**-**A** and **Mn**–**B**, and (C) relative LUMOs. Energies in parentheses are in kcal/mol. Angles (values in °) are defined by atoms highlighted in yellow.

Visual inspection of geometry-optimized **Mn**-**A** reveals that the metal ion deviates of 3° from the planarity of the square base defined by the Nε atoms of histidine residues and by the O of glutamine, mainly due to a slight shift of Q and H4 (O_Q_–Mn–N_H3_ and N_H1_–Mn–N_H4_ angles of 130° and 173° respectively, see Figure 2A).

A constrained geometry optimization was later carried out fixing the O_Q_–Mn–N_H3_ angle at 173°, aimed to evaluate the energy penalty for pushing Mn­(II) in the plane (**Mn**–**B** in Figure 2A lying at +1.8 kcal/mol on **Mn**-**A**).

This species turns back to **Mn**-**A** when the restraint is removed. These outcomes indicate that in the resting state, Mn­(II) is placed out of the plane generated by the histidine residues, in line with the experimental observation.^17,18^

Regarding the population of molecular orbitals, deviations from planarity influence the distribution of d-character in the LUMO of Mn­(II)-containing systems. In particular, for **Mn**-**A** and **Mn**–**B**, the LUMO characterized by *d*_3*z*_^2^_– *r*_^2^-π_Q/H_* can be observable for the former, while *d*_*xy*_-π_Q_* for the latter. More interestingly, the LUMO of **Mn**-**A** is 0.15 eV lower than that of **Mn**–**B**, corresponding to 3.5 kcal/mol. This suggests that with the out-of-plane geometry, the Lewis acidic nature of Mn­(II) is enhanced with the binding and activation of **Bnz** further increased. This aspect is also supported by the analysis of the Mulliken charges on the metal center, more positive in the case of **Mn**-**A** species (0.0 |e| vs −0.2 |e| in **Mn**–**B**). In addition, calculations of *f* ^+^ Fukui parameters for Mn in both systems, using finite difference approximation as implemented in UCA-Fukui,^41^ showed a slightly enhanced electrophilicity for **Mn**-**A** species (see Table S2).

Intriguingly, when the **Mn**-**A** model is optimized with Mn­(III), the relaxed geometry optimization returns to a cation located approximatively in the ligands’ square plane as **Mn**–**B** (ca 170°, see Figure S2), further corroborating the EPR results and the preference for Mn­(II) in the *Gt*HNL’s active site. This is additionally in line with the HSAB principle, since the softer nature of Mn­(II) with respect to Mn­(III) leads to better interactions with intermediate ligands such as imidazole from histidine or the carbonyl oxygen from glutamine.

### Binding Modes of Benzaldehyde and Cyanic Acid

With the distorted Mn­(II) character confirmed in the reduced model, the larger active-site model was applied to study the natural reaction and the enantioselectivity of *Gt*HNL. A number of enzyme–substrate (ES) complexes were optimized to identify the lowest-energy binding mode of **Bnz** and **HCN**. In accordance with the orientation of **Bnz** and with the location of **HCN**, it is possible to define pro-*R* and pro-*S* pathways. When the hydrogen of **Bnz** carbonyl group points toward T50, **HCN** can lay at the *Si* face (in the V42 direction) or at the *Re* face (in the V118 direction) of benzaldehyde. Attack from the *Si* face leads to the formation of *R*-product, while *S*-product is formed when cyanide attacks from the *Re* face (named **ES**-*R* ^**T50**/**V42**^ and **ES**-*S* ^**T50**/**V118**^, respectively; see Figure 3). In the case of aldehyde hydrogen pointing in the direction of Q59, the **HCN**, lying at the *Si* face (in the V118 direction) or at the *Re* face (in the V42 direction) of the substrate, leads to the formation of *R*-product and *S*-product, respectively (**ES**-*R* ^**Q59**/**V118**^ and **ES**-*S* ^**Q59**/**V42**^, see Figure 3). Therefore, these four different enzyme–substrate complexes (Figure 3) were geometry-optimized, with conformations of the active site residues resembling the crystal structure, as can be seen from the superposition illustrated in Figure S3.

**Figure 3:**
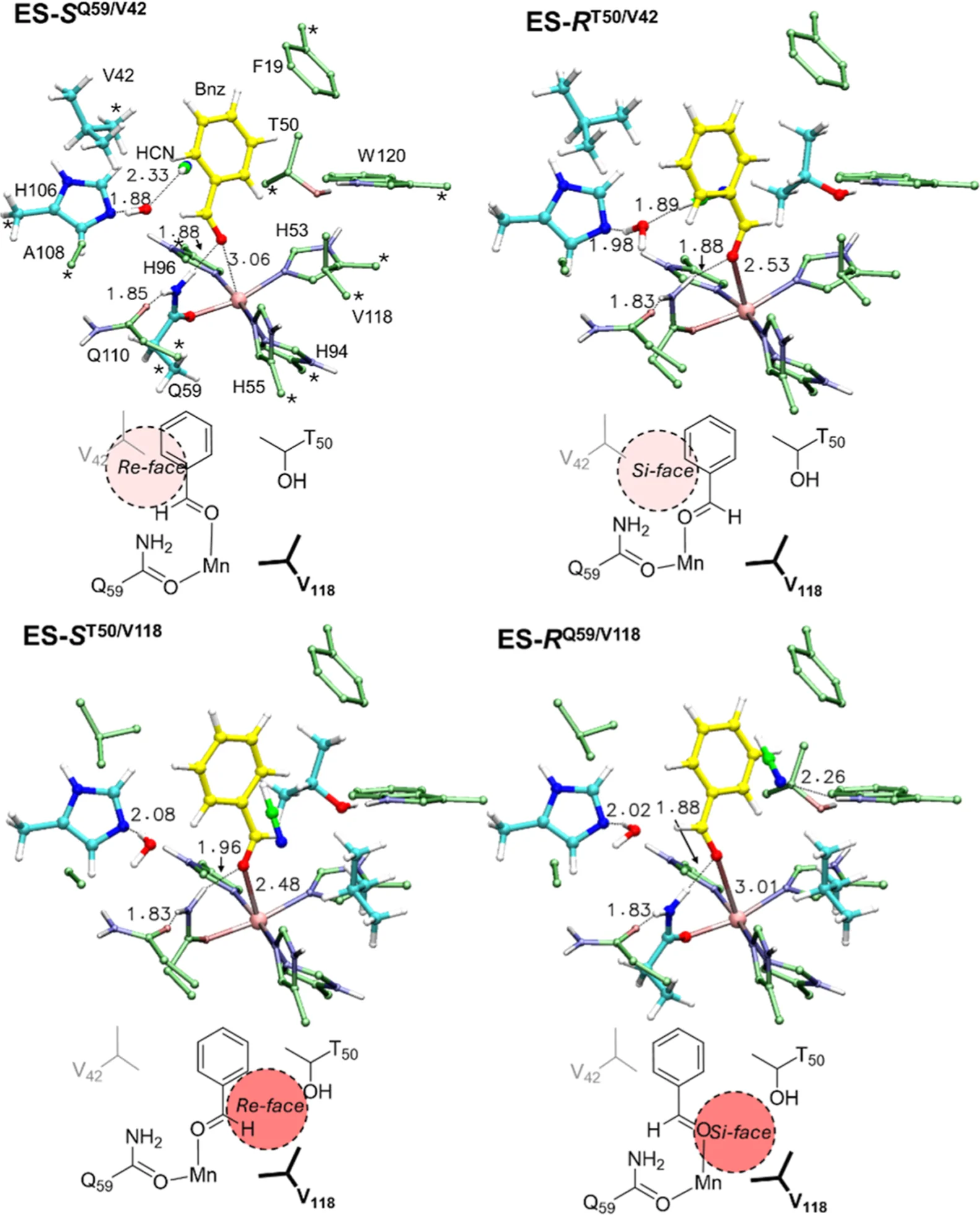
Optimized structures of the enzyme–substrate complexes and relative *Si* and Re faces of benzaldehyde in the *Gt*HNL active site. Circled areas indicate the binding region of hydrogen cyanide. Selected distances are given in Å. For clarity, most of the hydrogens are omitted. Atoms kept fixed during the geometry optimizations are labeled with an asterisk.

Interestingly, the complexes are very similar in energy despite looking structurally somewhat different, in a range of 3.6 and 4.2 kcal/mol, in protein and water environments, respectively.

**Bnz** occupies the vacant coordination site of Mn­(II) (O_Bnz_-Mn = 2.48 to 3.06 Å) and interacts with Q59 via hydrogen bond (O_Bnz_-H_Q59_ = 1.88 to 1.96 Å), while the aldehyde hydrogen is oriented toward Q59 or T50 (see Figure 3). In the lowest-energy structure **ES**-*S* ^**Q59**/**V42**^, **HCN** is accommodated in the pocket near V42, establishing a water-mediated hydrogen bond with H106 (see Figure 3). This complex would, however, yield the wrong enantiomer. In the case of lowest-energy pro-*R* structure **ES**-*R* ^**Q59**/**V118**^, **Bnz** coordinates Mn­(II) in a similar fashion to **ES**-*S* ^**T50**/**V118**^, while **HCN** lies in the V118 pocket and interacts with W120 via a hydrogen bond (N_HCN_-H_W120_ = 2.26 Å, see Figure 3). With almost identical energy, **ES**-*R* ^**T50**/**V42**^ with the correct orientation of the **HCN** close to the base H106 would match earlier experimental results. The **ES**-*R* ^**T50**/**V42**^ and **ES**-*S* ^**T50**/**V118**^ complexes lie at +1.5 and +3.6 kcal/mol (+1.9 and +4.2 kcal/mol in water environment), respectively, above **ES**-*S* ^**Q59**/**V42**^, and are both characterized by aldehydic H of **Bnz** in the direction of amino acid T50 and a shorter distance of O_Bnz_-Mn (2.53 Å and 2.48 Å, respectively, see Figure 3). In addition, the analysis of structural parameters of Mn­(II) coordination sphere in the four ES complexes, such as ligand–metal distances or angles of opposite corner points, reveals a good agreement with those arising from the available crystallographic structure (see details in Figure S4).^4,23^

From these four enzyme–substrate complexes, only one exhibits HCN closely aligned with the base H106 and the **Bnz** oriented such that *R*-**Mnd** will be formed, suggesting that substrates can move or shift in the active site for the occurrence of the reaction. In general, the energy obtained for optimized ESs indeed indicates that such movements can occur, with a small energy penalty. In the case of **HCN**, for instance, up to 2.1 kcal/mol are required, when compared with the relative energies of complexes having the same orientation of the species (see **ES**-*R* ^**T50**/**V42**^ vs **ES**-*S* ^**T50**/**V118**^ or **ES**-*S* ^**Q59**/**V42**^ vs **ES**-*R* ^**Q59**/**V118**^ in Figure 3). This is in agreement with the investigation of the mechanism of *Hb*HNL, for which it had been shown that **HCN** binds after the carbonyl compound (acetone in this case) and can move around it. The bound acetone displayed significant motility without a damaging energy toll.^11^ However, this raises the question whether a similar flexibility can also play a role in the *Gt*HNL catalysis and in particular in the rotation of **Bnz** around the Mn–O coordinative bond. Hence, starting from lowest-energy **ES**-*S* ^**Q59**/**V42**^, the mobility of **Bnz** in the active site model has been evaluated, studying the potential energy surfaces obtained for the rotation of the substrate around the Mn–O_Bnz_ bond, as shown in Figure S5.

Analysis of the results revealed that upon formation of the bond with the metal, **Bnz** has some flexibility to change its orientation in the active site. Indeed, a number of possible orientations in a range of 10 kcal/mol could be obtained in the active site by constraining the dihedral angle (see Figure S5), characterized by much lower energy than that required for the rate-determining step of the reaction as discussed later. The orientations assumed by the substrate are mainly accompanied by the shifts of hydrophobic residues such as F19 and A108 and by Q110 compatible with their frozen atoms. However, when the constraint is removed, the full geometry optimizations generated **ES**-*R* ^**Q59**/**V118**^- and **ES**-*S* ^**Q59**/**V42**^-like orientations. Furthermore, **Bnz**’s tendency to bind *Gt*HNL either in **ES**-**R** ^**Q59**/**V118**^- and **ES**-**S** ^**Q59**/**V42**^ was observed in 2 × 100 ns molecular dynamics (MD) simulations on the *Gt*HNL/**Bnz** system, as reported in Figure S6. In detail, this has been pinpointed by the analysis of distances between **Bnz** and Q59 or T50, that the substrate, prior to the binding to Mn­(II), lies in the enzyme’s catalytic pocket at ca. 8–10 Å for both distance pairs (see Figure S6). MD simulations further allowed the identification of solvent molecules in proximity of Mn­(II), confirming the solvent-exposed nature of enzyme’s active site (see first and second hydration spheres resulted calculation of radial distribution function for Mn­(II)-O_water_ pair in Figure S6).

Overall, these observations allow two pathways leading to *R*-**Mnd** and two to *S*-**Mnd**, all four without any rotation of the electrophile or shift of the nucleophile. Additionally, mixed pathways of the two *R*-**Mnd** and two to *S*-**Mnd** pathways could arise as will be discussed in the next sections (see Figure S7).

### Reaction Mechanism and Enantioselectivity of *Gt*HNL

The investigated reaction mechanism proposes that, upon the formation of the O_Bnz_-Mn bond in **ES**, **HCN** can undergo deprotonation by the general base H106 in **TS1** to generate cyanide species in **I1** (see Figure 4A).

**Figure 4:**
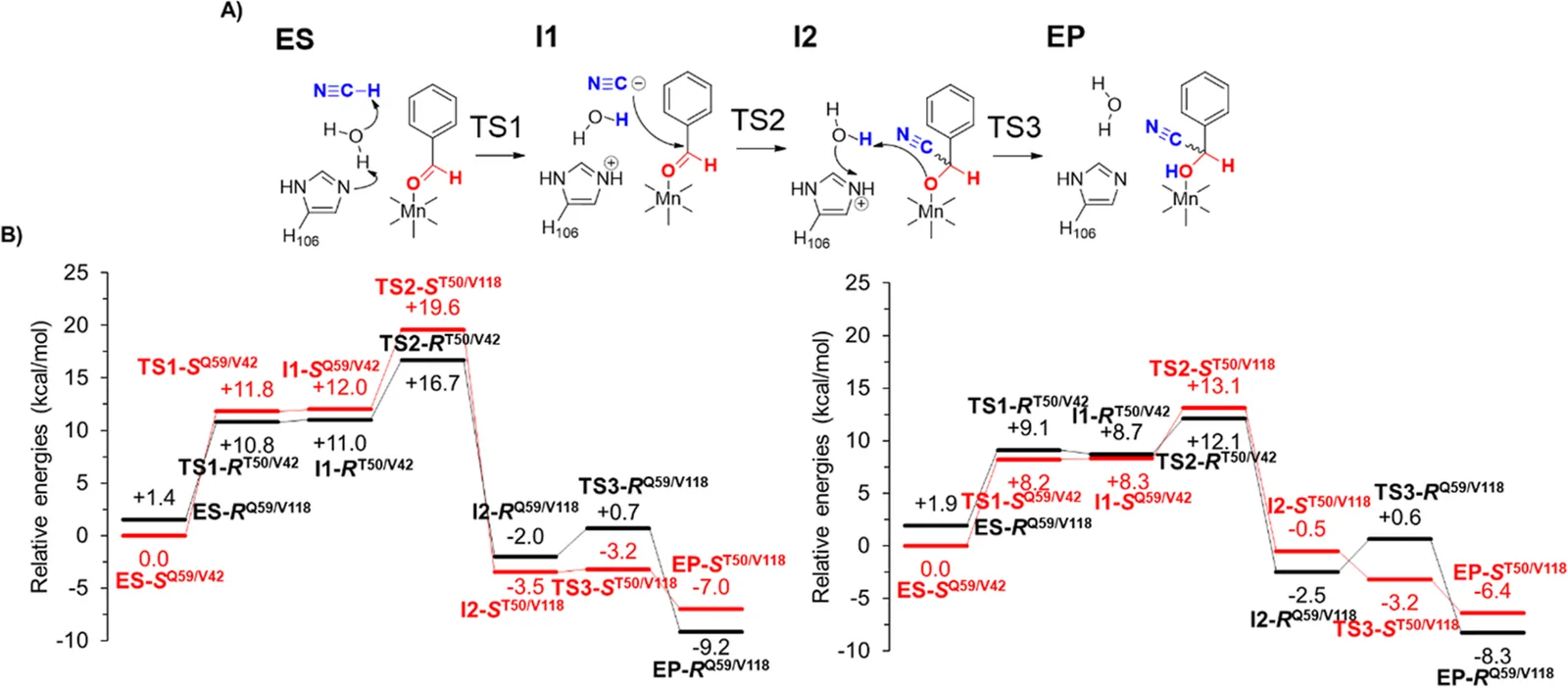
(A) Reaction mechanism of *Gt*HNL and (B) calculated lowest-energy profiles for the formation of mandelonitrile, in protein and water (bottom-left and bottom-right, respectively). Relative energies concern final electronic energies, corrected with solvation and ZPE energies (see the Technical Details section for specifics).

Cyanide can thus attack the prochiral C atom of the **Bnz** carbonyl group (**TS2**) to form deprotonated mandelonitrile (**Mnd**) in **I2**. Finally, the protonated H106 can deliver, in a water-mediated fashion (**TS3**), the H^+^ to the product with the formation of neutral **Mnd**, thus restoring its catalytically active state (**EP**, see Figure 4A).

Each ES among the four complexes depicted in Figure 3 was taken into consideration to investigate the reaction mechanism and the enantioselectivity. As a result, intermediates and transition states were geometrically optimized, starting from **Bnz** and **HCN** oriented as stated in the paragraph above. Figure 4B displays the lowest-energy profiles that were generated for pro-*R* and pro-*S*, while the others are shown in Figure S8. In **TS1**-*R* ^**T50**/**V42**^ and **TS1**-*S* ^**Q59**/**V42**^, the energy amount for proton-shuttled activation of **HCN** by H106 corresponds to 10.8 and 11.8 kcal/mol in protein and +9.1 kcal/mol and +8.2 kcal/mol in water, respectively. The activated **HCN** (**CN** ^–^) is thus formed in **I1**-*R* ^**T50**/**V42**^ and **I1**-*S* ^**Q59**/**V42**^, at +11.0 kcal/mol and +12.0 kcal/mol in protein and at +8.7 kcal/mol and +8.3 kcal/mol in water, respectively (see Figure 4B for energies and Figure S9 for optimized geometries).

In the case of the investigated pro-*R* pathway, during the transition state, **HCN** moves from the V118 pocket to V42, getting in proximity of H106 for the activation concertedly to the rotation of the **Bnz** substrate, in analogy to the calculations discussed above and to the experimental outcomes for the *Hb*HNL.^11^ This reorganization is necessary for proceeding with the reaction scheme of Figure 4A, considering that in the V118 direction, there are no basic amino acid residues able to enhance **HCN** nucleophilicity through its deprotonation. This preorganization of the active site is further confirmed by analysis of Mulliken and Hirshfeld charges in the ESs, which clearly highlights a more negative charge in proximity to N_H106_ (see Table S3). In addition, the **HCN** activation step becomes mandatory since preliminary attempts to find transition states without deprotonation of **HCN** or with concerted water-mediated deprotonation by H106 resulted in the respective ESs that ruled this mechanistic route. After deprotonation, the nucleophilicity of C_HCN_ is further enhanced, as confirmed by calculated charges (see **ES** vs **I1** values in Table S3). Due to the closeness of energy values, however, we cannot exclude that **Bnz** and **HCN** can be in a **TS1**-*R* ^T50/V42^-like conformation, thus avoiding shift or rotation of the substrates in the active site.

In both lowest-energy intermediates **I1**, the negatively charged **CN** ^–^ group is kept in proximity of **Bnz** (C_CN_-C_Bnz_ = 2.93 Å, see Figure S8), lying in V42-direction and in a productive orientation for the proceeding of the reaction. In the case of the pocket in proximity of V118, after the geometry-optimization, **CN** ^–^ resulted in hydrogen bonds with W120 and Q59 in both pro-*R* and pro-*S* routes (see **I1**-*S* ^**T50**/**V118**^ and **I1**-*R* ^**Q59**/**V118**^ Figure S9). However, the energy of the intermediates resulted ca 9 kcal/mol higher than the respective lower-energy ones considered in the energy profiles of Figure 4B.

The C–C bond formation is observed in the second stage of the reaction, where C_CN_ approaches C_Bnz_ at 2.37 Å and 2.43 Å, in the case of pro-*S* and pro-*R* pathways, respectively (see **TS2**-*S* ^**T50**/**V118**^ and **TS2**-*R* ^**T50**/**V42**^ in Figure 5).

**Figure 5:**
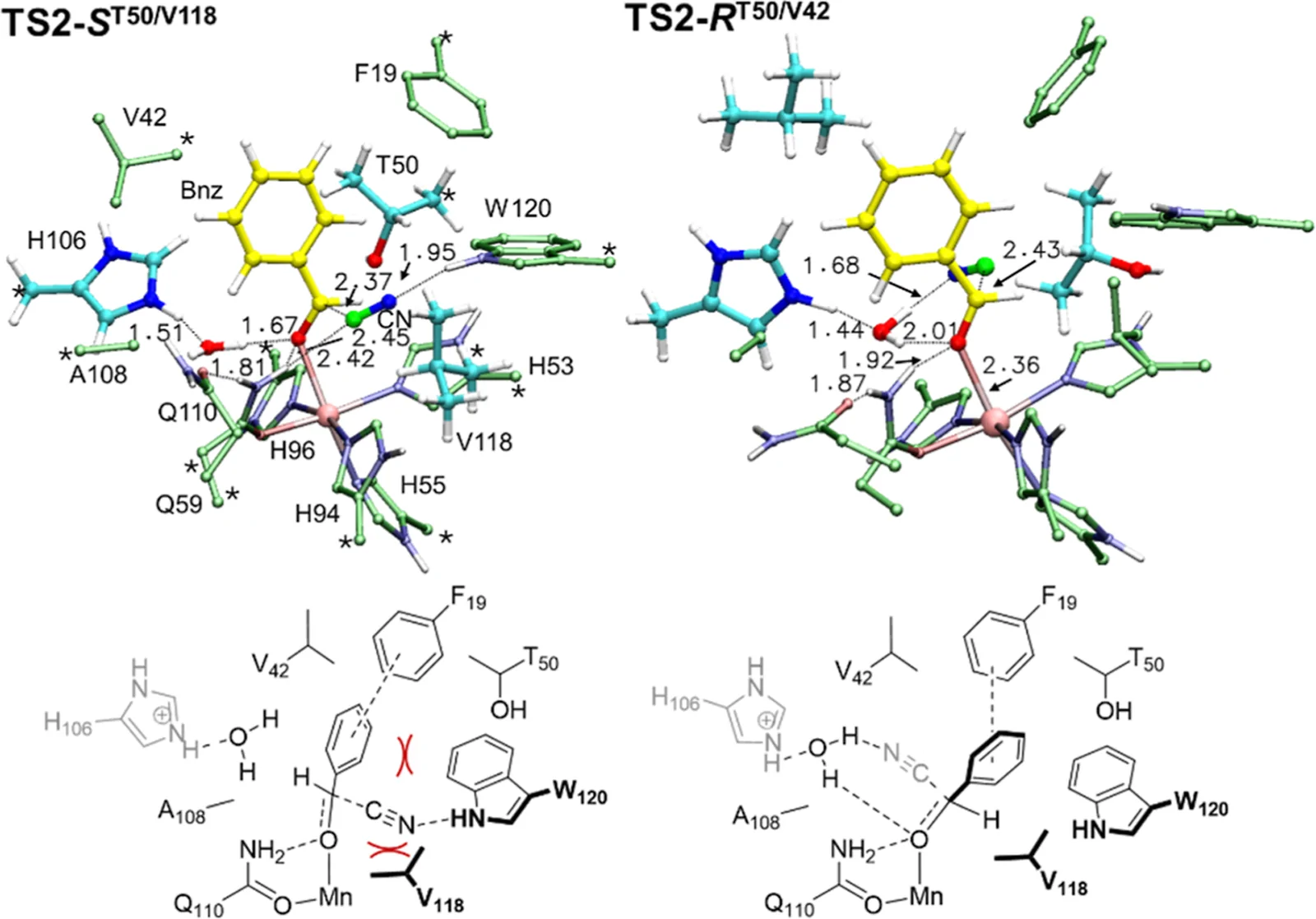
Optimized structures of lowest-energy transition states **TS2**-*S* ^**T50**/**V118**^ and **TS2**-*R* ^**T50**/**V42**^. Selected distances are given in Å. For clarity, most of the hydrogens are omitted. Atoms kept fixed during the geometry optimizations are labeled with an asterisk.

The closer view of the optimized transition states reveals that **CN** ^–^ “nucleophile” attacks the carbon atom of the aldehyde group from V118- and V42-direction, thus characterizing **TS2**-*S* ^**T50**/**V118**^ and **TS2**-*R* ^**T50**/**V42**^, respectively. The position of **CN** ^–^ therefore generates evident differences in the two transition states, as can be evidenced by visual inspection of the optimized structures (Figure 5). Looking at this figure emerges indeed that, in the case of **TS2**-*S* ^**T50**/**V118**^, the **Bnz** suffers clashes between its aromatic ring and W120, while **CN** ^–^ causes a shift of V118 due to steric hindrance. The carbon atom of cyanide, in addition, interacts with the amide group of Q59, elongating the O_Bnz_-H_Q59_ hydrogen bond destabilizing the transient oxyanion species in the transition state. Furthermore, noncovalent interaction plot of the TSs evidence that the T-shaped π–π interaction involving F19 and **Bnz** is weaker in the case of the pro-*S* transition state, due to the orientation of substrate’s aromatic ring.

The consequent destabilization of **TS2**-*S* ^**T50**/**V118**^ is quantified in 2.9 and 1.0 kcal/mol in protein and water environments, respectively, compared to **TS2**-*R* ^**T50**/**V42**^ (19.6 kcal/mol vs 16.7 and 13.1 kcal/mol vs 12.1 kcal/mol with respect to relative **ES**-*S* ^**T50**/**V118**^ in protein and water, respectively, see Figure 4B). The transition-state energy differences in both media are consistent with the stereoselectivity observed for the enzyme in favor of the *R*-enantiomer (86% ee).^11^ Transition states **TS2**-*S* ^**Q59**/**V42**^ and **TS2**-*R* ^**Q59**/**V118**^ were found up to 5 kcal/mol higher than **TS2**-*S* ^**T50**/**V118**^ and **TS2**-*R* ^**T50**/**V42**^, respectively (see the optimized structures in Figure S10). In addition to the proper rationalization of enantioselectivity in terms of visual inspection/detection of shifts caused by different binding modes of the substrates, single-point energy calculations were further performed to quantify the contribution of F19/Q59/V118/W120 to **ES**-**S** ^**Q59**/**V42**^, **TS2**-**R** ^**T50**/**V42**^, and **TS2**-**S** ^**T50**/**V118**^ using the counterpoise procedure (see further details in Table S4).^42^ To do so, starting from the optimized structures and in analogy to recent work,^43^ affinities for **Bnz** and **HCN** were compared between the full model and those obtained excluding that single amino acid (F19/Q59/V118/W120 in this case) in the stationary points. The main stabilization occurs in **TS2**-**S** ^**T50**/**V118**^ by W120 and Q59 (ca. 10 kcal/mol, see Table S4), which are involved in hydrogen bond interactions with **CN** ^–^. A slighter effect can be noticed for **TS2**-**R** ^**T50**/**V42**^, since both **Bnz** and **HCN** are not in proximity to the considered amino acid residues, as in the case of **TS2**-**S** ^**T50**/**V118**^.

The deprotonated *R*-**Mnd** and *S*-**Mnd** are later formed in the respective **I2**-*R* ^**Q59**/**V118**^ and **I2**-*S* ^**T50**/**V118**^ intermediates, lying at −2.0 kcal/mol and −3.5 kcal/mol in protein and −2.5 kcal/mol and −0.5 kcal/mol in water below the reactant, respectively (see energies in Figure 4B and optimized structures in Figure S11). As discussed above, for this to occur, a rotation of the ligand around the Mn–O bond is required. Accordingly, the associated energy penalty for rotating the deprotonated **Mnd** species was investigated, starting from the lowest-energy **I2**–**S** ^**T50**/**V118**^ structure by constraining the relevant dihedral angle (see Figure S12). It turned out that, similarly to the study of **Bnz** conformations in ES, such rotation can take place in a range of ca 10 kcal/mol resulting lower than the energy barrier calculated for the rate-determining step of the reaction. In addition, when rotating, the deprotonated **Mnd** causes a slight shift of F19, T50, and V118, that can adapt to the movement of the ligand. According to the calculations, the final step of the reaction takes place with very fast kinetic through **TS3**-*R* ^**Q59**/**V118**^ and **TS3**-*S* ^**T50**/**V118**^, with energy barriers within *ca* 3 kcal/mol from relative intermediates **I2**, see Figures 4B and S13. At this stage, the initial catalytic state of H106 is restored by the water-mediated H^+^ delivery to the product, and the neutral *R*-**Mnd** and *S*-**Mnd** are finally obtained in **EP**-*R* ^**Q59**/**V118**^ and **EP**-*S* ^**T50**/**V118**^.

Rooms in proximity to V42 are generated by small rotations of the amino acid residue to accommodate the aromatic ring of **Mnd**, while the cyanide moiety is settled in the direction of V118, with a slight shift of W120 in the case of the pro-*S* route (see Figure 6).

**Figure 6:**
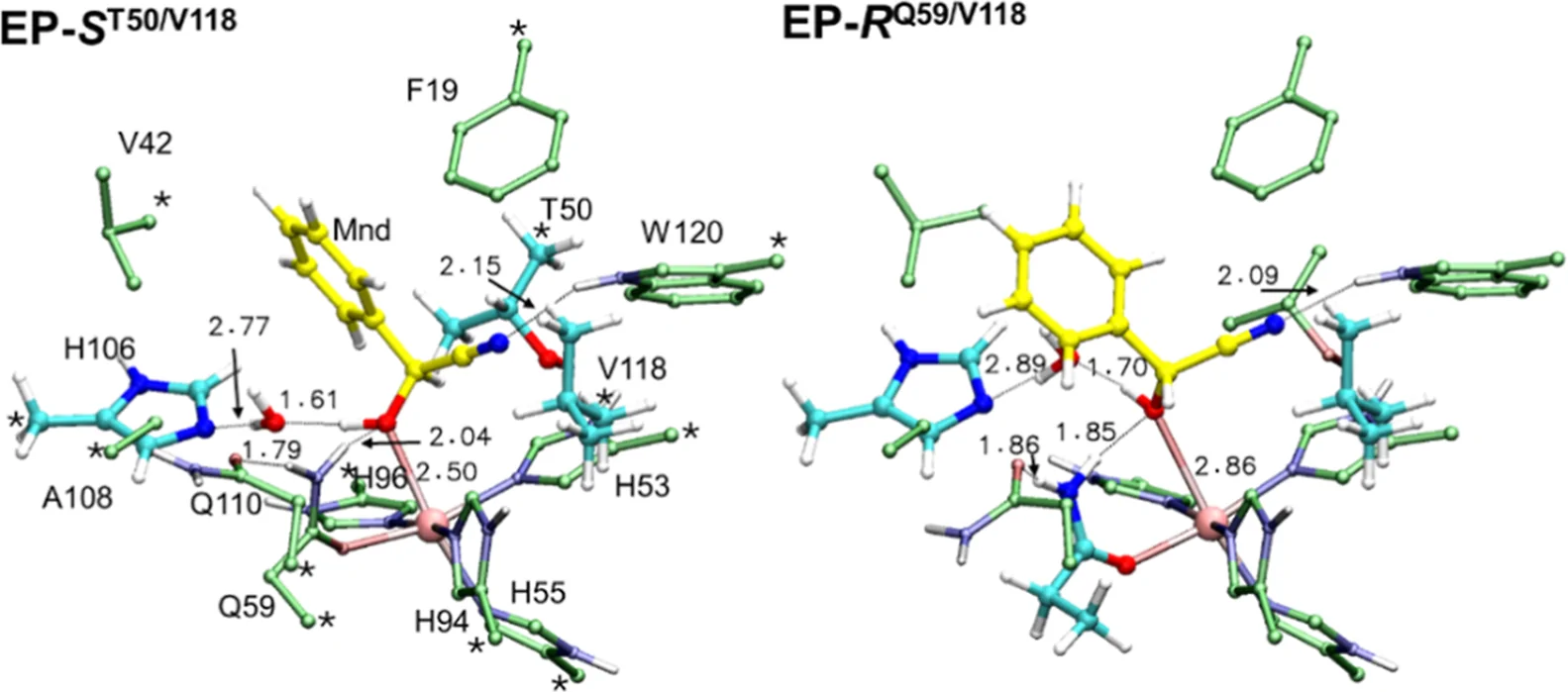
Optimized structures of lowest-energy **EP**-*S* ^**T50**/**V118**^ and **EP**-*R* ^**Q59**/**V118**^. Selected distances are given in Å. For clarity, most of the hydrogens are omitted. Atoms kept fixed during the geometry optimizations are labeled with an asterisk.

However, despite the structural differences, favorable thermodynamics is observed for both products (7.0 and 9.2 kcal/mol in protein and 6.4 and 8.3 kcal/mol in water, below **ES**-*S* ^**Q59**/**V42**^ for *S*-**Mnd** and *R*-**Mnd** respectively, see Figure 4B). Other EP complexes were considered, resulting in higher energy of *ca* 7 kcal/mol relative to the lowest-energy ones (see Figure S14). **TS2**-*R* ^**T50**/**V42**^ has been identified as the rate-limiting step of the reaction leading to *R*-**Mnd**. Its energy barrier of 16.7 and 13.1 kcal/mol, in protein and water environments, respectively, is in agreement with the available experimental *k*_*cat*_ of 0.075 s^–1^, measured at pH 5.5, that corresponds to 18.6 kcal/mol at room temperature.^14^ Moreover, as **Bnz** is the reference substrate for HNL’s additional data, HNLs from several other organisms are available with turnover numbers ranging in 30–3000 s^–1^.^44–46^ These values correspond to activation energies of 15–12 kcal/mol, well covering the calculated barriers.

Care has to be taken with all reported *k*_*cat*_ and *K*_*m*_ *’* values of the enzymatic cyanohydrins formation, as all experimental data were acquired with different systems with structurally unrelated enzymes.

Overall, the energetic and structural analysis highlights that *Gt*HNL favors the attack of **CN** ^–^ to **Bnz** in the V42-side of the active site, where hydrogen cyanide/cyanide can be engaged in hydrogen-bond networks, while in V118, the deprotonated/neutral **Mnd** can be accommodated. The active site can adapt to the presence of both substrates, as highlighted by distance values reported in Table S5.

As expected, differences arising from the comparison between energetic profiles in protein and water environments can be noted. This can be related to the sensitivity of different dielectric constants on the distribution of charge during the mechanism investigated. Importantly, although the absolute values varied, the relative energies remained unchanged, thereby preserving the shape of the potential energy surface. Consequently, the choice of the environment did not affect the conclusions.

The role of *Gt*HNL was further highlighted by the study of **Mnd** synthesis in solution, i.e., in the absence of any catalysts. In particular, from the analysis of the results in Figure S15, it is clear how the reaction in the presence of the enzyme occurs faster, due to the decreasing of the activation energy barrier of *ca* 15 kcal/mol (33.1 kcal/mol in solution vs 16.7 kcal/mol in the enzymatic environment, see Figure S16). This effect is mainly due to the presence of Mn­(II), which stabilizes the transition state and activates the C = O_Bnz_ of the substrate for the attack of **CN** ^–^ along the Bürgi–Dunitz angle, in agreement with the previous hypothesis (see Figure S16).^17,18^

Overall, it is important to underline that any contribution to the reactivity and to the enantioselectivity by the formation of the ES or the release of the product from the active site was not considered explicitly for *Gt*HNL with the adopted strategy. This is based on the assumption that they do not have an impact on the reaction’s rate (determined by the chemical step) and on the enantioselectivity (expression of enantiomeric excess (*ee*) is dependent on the activation energies of lowest-energy pro-*R* and pro-*S* transition states).^47^As a final remark, interestingly, the calculations show that the energy differences obtained for the ES complex (in favor of pro-*S* pathway) do not correspond to those observed for both lowest-energy transition states and EP complexes (lying on the pro-*R* route). The results indicate that, for *Gt*HNL as for other enzymes,^47,48^ the stereochemistry can be accurately predicted and explained by looking at the full energy profile of the reaction mechanism and not by focusing partially on the ES complex.

### Effect of Mutations on the Reactivity and Enantioselectivity of *Gt*HNL

To gain deeper insight of *Gt*HNL, the effect of mutations on its reactivity was further investigated. Mutations of bulky and aromatic residues, either F19A or W120A, were considered, and six new transition states were geometry-optimized, starting from the previously discussed ones, upon replacement of either benzyl or indole rings of F19 or W120 by a methyl group, respectively. Results show that, in the presence of both F19A and W120A mutations, the relative activation energy Δ*E* ^‡^ of the pro-*R* and pro-*S* transition states is in favor of **TS2**_**F19A**_-*R* ^**T50**/**V42**^ and **TS2**_**W120A**_-*S* ^**T50**/**V42**^, up to 6.4 and 16.2 kcal/mol, respectively (Figures S17 and S18), as observed for the wildtype *Gt*HNL and summarized in Table 1. In the case of **TS2**_**F19A**_-*R* ^**T50**/**V42**^, the substrate has more room to slightly rotate and straighten the T-shaped π–π interaction with W120, stabilizing the transition states more than those of the pro-*S* pathway (which suffer of similar steric clashes as in the case of the wilt-type model, see Figure S17). In the case of W120A, a closer look to the transition state of **TS2**_**W120A**_-*S* ^**T50**/**V42**^ reveals that the empty space generated by the mutation is occupied by the **Bnz** substrate, favoring a proper π–π interaction with F19 (see Figure S18). The calculations suggested that the inversion of enantioselectivity, i.e., formation of *S*-**Mn** **d**, is elusive focusing on these single mutations, or the effect of mutating residues is not fully caught by the current modeling strategy.

**Table 1:** Summary of Activation Energies, in a Protein Environment, Calculated for Lowest Energies Transition States in the Investigated Mutation-Containing Systems^a^

systems	Δ*E* ^‡^	ΔΔE^‡^ (*S*–*R*)
*Gt*HNL	16.7 [13.1]	2.9 [1.0]
*Gt*HNL_F19A_	-	4.5 [2.9]
*Gt*HNL_W120A_	-	2.2 [1.9]
*Gt*HNL_mut_	20.6 [18.8]	–0.2 [+0.1]

a In square brackets, values in water are reported. All values are in kcal/mol.

Considering experimental evidence on *Thermotoga maritima* 1010 species (*Tm*1010), an enzyme that holds in its active site most of the amino acid residues as *Gt*HNL and that exhibits a negligible hydroxynitrile lyase activity,^13^ the impact of the different amino acids on the catalytic activity of *Gt*HNL was investigated. In detail, the mutations A108G, V118A, and Q110S were included in the current model, and new geometry optimizations of the four ES complexes were carried out, in addition to that of the lowest-energy pro-*R* and pro-*S* transitions state. Analogously to the wildtype model, the **HCN** substrate is accommodated in a pocket near V42, with aldehydic hydrogen pointing Q59 in lowest energy **ES**_**mut**_-*S* ^**Q59**/**V42**^ and with other ESs ranging in *ca* 4 kcal/mol (see Figure S19). For the optimized **TS2**_**mut**_-*S* ^**T50**/**V118A**^ and **TS2**_**mut**_-*R* ^**T50**/**V42**^, very similar energy resulted, being at +20.6 kcal/mol vs +20.8 kcal/mol in protein and +18.8 vs +18.9 kcal/mol in water for pro-*S* and pro-*R* pathways, respectively, with respect to **ES**_**mut**_-*S* ^**Q59**/**V42**^ (optimized structures in Figure S19). Geometry inspection shows that A108G, V118A, and Q110S generate rotations of species in the active site, due to reorganization of the hydrogen-bond network, as in the case of Q110S-Q59 interaction, or due to new pockets occupied by **Bnz** and **CN** ^–^, as in the case of A108G and V118A. These results can be linked to the weaker HNL activity of *Tm*1010 previously commented.^13^ The activation energy was calculated to be 3.9 and 6.4 kcal/mol higher than wildtype relative transition state energies in protein and water environments, respectively (corresponding to lowering reaction rate by 10^3^–10^5^ fold at room temperature, see Table 1). Moreover, with the obtained energy difference between **TS2**_**mut**_-*S* ^**T50**/**V118A**^ and **TS2**_**mut**_-*R* ^**T50**/**V42**^, the enantiopreference is loss. Due to the nature of mutated residues, it could be hypothesized that smaller residues, such as A108G and V118A, make a larger/more solvent-exposed *Tm*1010s active site, where **Bnz** or **HCN** could miss the binding of the enzyme, thus justifying the weaker HNL activity of the enzyme.

## Conclusions

In the present study, we have adopted the QM-cluster model in the framework of DFT to investigate many aspects concerning the reaction mechanism of hydroxynitrile lyase from *Granulicella tundricula*. The enzyme can catalyze the synthesis of *R*-mandelonitrile from benzaldehyde and hydrogen cyanide with good enantioselectivity, and its use is of interest for further biocatalytic applications.

The contribution of Mn­(II) to the catalysis was elucidated, and, more detailed, the role of the out-of-plane conformation of the cation was rationalized in terms of enhancement of metal Lewis acidity. Calculations further corroborate previous EPR studies, ruling out the presence of Mn­(III) over Mn­(II) in the active site.

The reaction mechanism has been investigated in protein and water environments, starting from a model of the enzyme active site obtained by the recently solved X-ray structure of *Gt*HNL, and, in general, the results showed a good agreement with the available kinetic data counterpart concerning the synthesis of *R*-mandelonitrile. The consideration of different conformations of benzaldehyde and hydrogen cyanide for the optimizations of intermediates and transition states was needed and allowed to pinpoint the origin of stereoselectivity. The benzaldehyde’s flexibility in the active site was highlighted and, more importantly, it was proposed that this is guaranteed by F19 and A108 residues that can adapt to the rotation of the substrate upon the binding to Mn­(II).

It has been demonstrated that the activation of hydrogen cyanide by H106 is a required step and occurs in the V42 direction of the active site, where the enzyme is preorganized to accommodate and deprotonate the substrate. Later, C–C bond formation between hydrogen cyanide and benzaldehyde can take place, and it is calculated as the rate-determining step of the reaction. Comparison with the results obtained from the study of the reaction in solution highlighted the role of the metal center at this step, which stabilizes the transient negative charge of the carbonyl group. Mandelonitrile is later obtained with favorable thermodynamics, with the cyanide group lying in the V118 side of the *Gt*HNL active site.

In summary, concerning the role of Mn­(II), electronic structures pinpointed on the contribution of metal’s atomic orbital to the formation of the initial enzyme–substrate complex, as well as quantified the stabilizing effect of transient oxyanion formed during the reaction mechanism.

Moreover, the calculations highlighted that, during the catalysis, benzaldehyde and hydrogen cyanide can rotate/move in the active site without high-energy penalty, with respect to the rate-determining step, and that these rotations/rearrangements favor the occurrence of the reaction through the lower-energy pro-*R* pathway. This is made possible by amino acid residues, like F19, A108, V118, and Q110, that minimize the impact of the rotation by adapting to it. A similar behavior can be assigned also to the deprotonated mandelonitrile.

The effect of single mutations F19A and W120A to the enantioselectivity was further investigated in silico, highlighting their role in orienting the benzaldehyde substrate for proper reactivity with *Gt*HNL. Calculations further suggest that single mutations of these bulky residues are not enough for inverting the enzyme’s enantiopreference. Moreover, considering A108G, V118A, and Q110S—present in *Tm*1010 species—in the model allowed the rationalization of their contribute to the negligible hydroxynitrile lyase activity of the enzyme, which could be related to an affected binding of substrates for the reaction.

Finally, the need for full energy profiles to predict/explain the enantioselectivity of *Gt*HNL was commented on. In particular, the energy differences between pro-*R* and pro-*S* of the intermediates and, more importantly, of the transition states suggest that it is not accurate to focus only on ES or EP complexes for stereochemical outcomes because these geometries can lack the energetic origins of enzymatic enantioselectivity.

## Supplementary Material





## Data Availability

The data supporting this article have been included as part of the SI.

## Notes

Cartesian coordinates of all optimized geometries in the PDB format (ZIP)
Additional results discussed in the text and absolute energies for all stationary points (PDF)
