# Supplementary material for: Activity and Enantioselectivity of Hydroxynitrile Lyase from Granulicella tundricula: Computational Insights
Source: Inorg Chem. 2026 Aug 22;65(35):20293–303. doi: 10.1021/acs.inorgchem.6c02524 (PMC13556127; doi:10.1021/acs.inorgchem.6c02524)
Supplement: Supplementary file 1 [file ic6c02524_si_002.pdf]

-Supporting information-

Activity and enantioselectivity of Hydroxynitrile Lyase from *Granulicella tundricula:* Computational Insights

Mario Prejanò,[a,*] Ulf Hanefeld,[b,*] Tiziana Marino[a]

[a]*Department of Chemistry and Chemical Technologies, University of Calabria, Via Pietro Bucci 14/C, 87036 Arcavacata di Rende, CS, Italy*

[b]*Biokatalyse, Afdeling Biotechnologie, Technische Universiteit Delft, Van der Maasweg 9, 2629 HZ Delft, The Netherlands*

*Corresponding authors: mario.prejano@unical.it; u.hanefeld@tudelft.nl

Table of content

Figure S1. Optimized structures of ES-$S^{Q59/V42}$ with multiplicities 2S+1=3/2 and 2S+1=1/2. Relative energies in parenthesis are indicated in kcal mol^{-1} and are referred to energy from geometry optimization of ES-$S^{Q59/V42}$ with multiplicity 2S+1=5. Selected distances are given in Å. For clarity, most of the hydrogens are omitted. Atoms kept fixed during the geometry optimizations are labeled with asterisk. S4
Figure S2. Optimized structure of Mn-A model with Mn(III), including first coordination sphere of the cation (Q=Q59; H1=H96; H2=H96; H3=H53; H4=H55). S4
Figure S3. Superposition of crystallographic structure and optimized ESs (transparent representation). S5
Figure S4. Comparison of relevant geometric parameters of Mn(II) coordination sphere of crystallographic structure and optimized ESs. S5
Figure S5. On the left, scanned dihedral angle for the study of Bnz flexibility in *Gt*HNL active site and relative relaxed energy profile. On the right, superposition of all the obtained structures. S6
Figure S6. Results from MD simulation on 2x100 ns of *Gt*HNL:Bnz system. Technical details on simulations are provided below. S6
Figure S7. Energy profiles calculated for the formation of mandelonitrile, in ε=4.0 and ε=78.3 (top and bottom respectively). S7
Figure S8. Optimized structures of lowest-energy transition states that lead to the enantiomers TS1-$S^{Q59/V42}$ and TS1-$R^{T50/V42}$. Selected distances are given in Å. For clarity, most of the hydrogens are omitted. Atoms kept fixed during the geometry optimizations are labeled with asterisk. S8
Figure S9. Optimized structures of I1 intermediates that lead to the enantiomers *S* and *R*. Selected distances are given in Å. For clarity, most of the hydrogens are omitted. Atoms kept fixed during the geometry optimizations are labeled with asterisk. S9
Figure S10. Optimized structures of TS2-$S^{Q59/V42}$ and TS2-$R^{Q59/V118}$. that lead to the enantiomers *S* and *R*. Selected distances are given in Å. For clarity, most of the hydrogens are omitted. Atoms kept fixed during the geometry optimizations are labeled with asterisk. S10
Figure S11. Optimized structures of I2 intermediates that lead to the enantiomers *S* and *R*. Selected distances are given in Å. For clarity, most of the hydrogens are omitted. Atoms kept fixed during the geometry optimizations are labeled with asterisk. S11
Figure S12. On the left, scanned dihedral angle for the study of deprotonated Mnd flexibility in *Gt*HNL active site and relative relaxed energy profile. On the right, superposition of all the obtained structures. S12
Figure S13. Optimized structures of TS3 transition states that lead to the enantiomers *S* and *R*. Reported distances are given in Å. For clarity, most of the hydrogens are omitted. Atoms kept fixed during the geometry optimizations are labeled with asterisks. S13
Figure S14. Optimized structures of EP-$S^{Q59/V42}$ and EP-$R^{Q59/V118}$. Selected distances are given in Å. For clarity, most of the hydrogens are omitted. Atoms kept fixed during geometry optimizations are labeled with asterisks. S14
Figure S15. Calculated energy profile for uncatalyzed formation of mandelonitrile. S14
Figure S16. Focus on geometrical parameters characterizing the lowest-energy TS2-$S^{Q59/V42}$ and TS2-$R^{T50/V42}$ and the transition state of the uncatalyzed reaction (TS_{uncat}). S15
Figure S17. Optimized structures of TS2 with F19A mutations that lead to the enantiomers *S* and *R*. Reported distances are given in Å. Relative energies in round and square parenthesis (for protein and water environment respectively) are indicated in kcal mol^{-1}. For clarity, most of the hydrogens are omitted. Atoms kept fixed during the geometry optimizations are labeled with asterisks. S15
Figure S18. Optimized structures of TS2 with W120A mutations that lead to the enantiomers *S* and *R*. Reported distances are given in Å. Relative energies in round and square parenthesis (for protein and water environment respectively) are indicated in kcal mol^{-1}. For clarity, most of the hydrogens are omitted. Atoms kept fixed during geometry optimizations are labeled with asterisks. S16

Figure S19. Optimized structures of ES complexes and TS2 with A108G, Q110S and V118A mutations that lead to the enantiomers *S* and *R*. Reported distances are given in Å. Relative energies in parenthesis are indicated in kcal mol^{-1}. For clarity, most of the hydrogens are omitted. Atoms kept fixed during geometry optimizations are labeled with asterisks. S17
Table S1. Spin densities on Mn^{2+}, calculated for all the optimized structures...............................S18
Table S2. Fukui parameter f^+calculated for Mn-A and Mn-B species (for clarity, results of hydrogen atoms were omitted). S18
Table S3. Hirshfield-CM5 charges calculated for optimized ES complexes (Mulliken population in parenthesis). S19
Table S4. Affinities for Bnz and HCN substrates in ES-SQ59/V42, TS2-RT50/V42 and TS2-ST50/V118, calculated via counterpoise method at SDD/B3LYP-D3/6-31G(d,p) level of theory. Values are in kcal/mol.. ....S19
Table S5. Relevant geometric parameters of optimized intermediates and transition states.. ....S21
Table S6. Calculated B3LYP-D3 absolute energies (in a.u.). Main imaginary frequency ($\tilde{\nu}$) of transition states is reported in cm^{-1}. S21
Techincal details for MD simulations S19

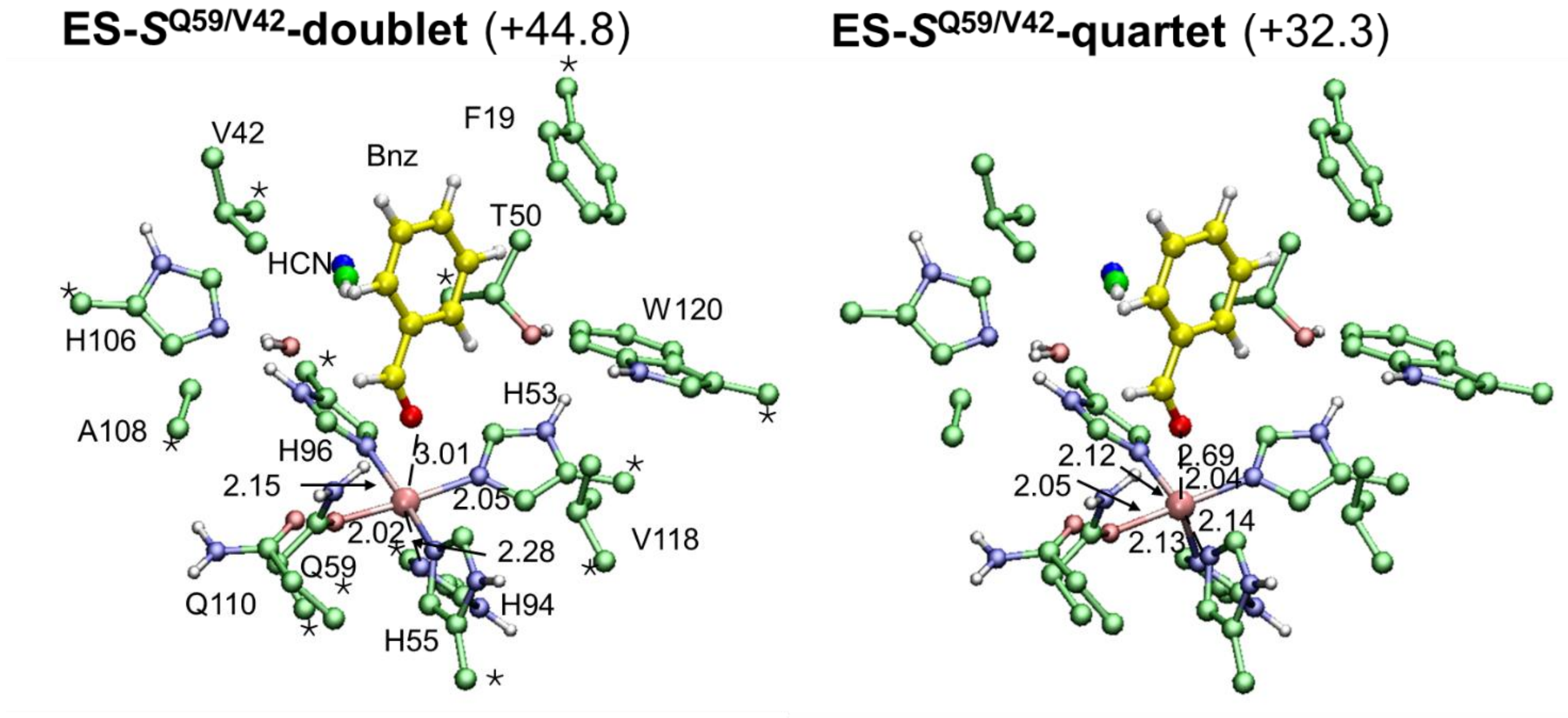

Figure S1. Optimized structures of **ES-*S*$^{Q59/V42}$** with multiplicities 2S+1=3/2 and 2S+1=1/2. Relative energies in parenthesis are indicated in kcal mol^{-1} and are referred to energy from geometry optimization of **ES-*S*$^{Q59/V42}$** with multiplicity 2S+1=5. Selected distances are given in Å. For clarity, most of the hydrogens are omitted. Atoms kept fixed during the geometry optimizations are labeled with asterisk.

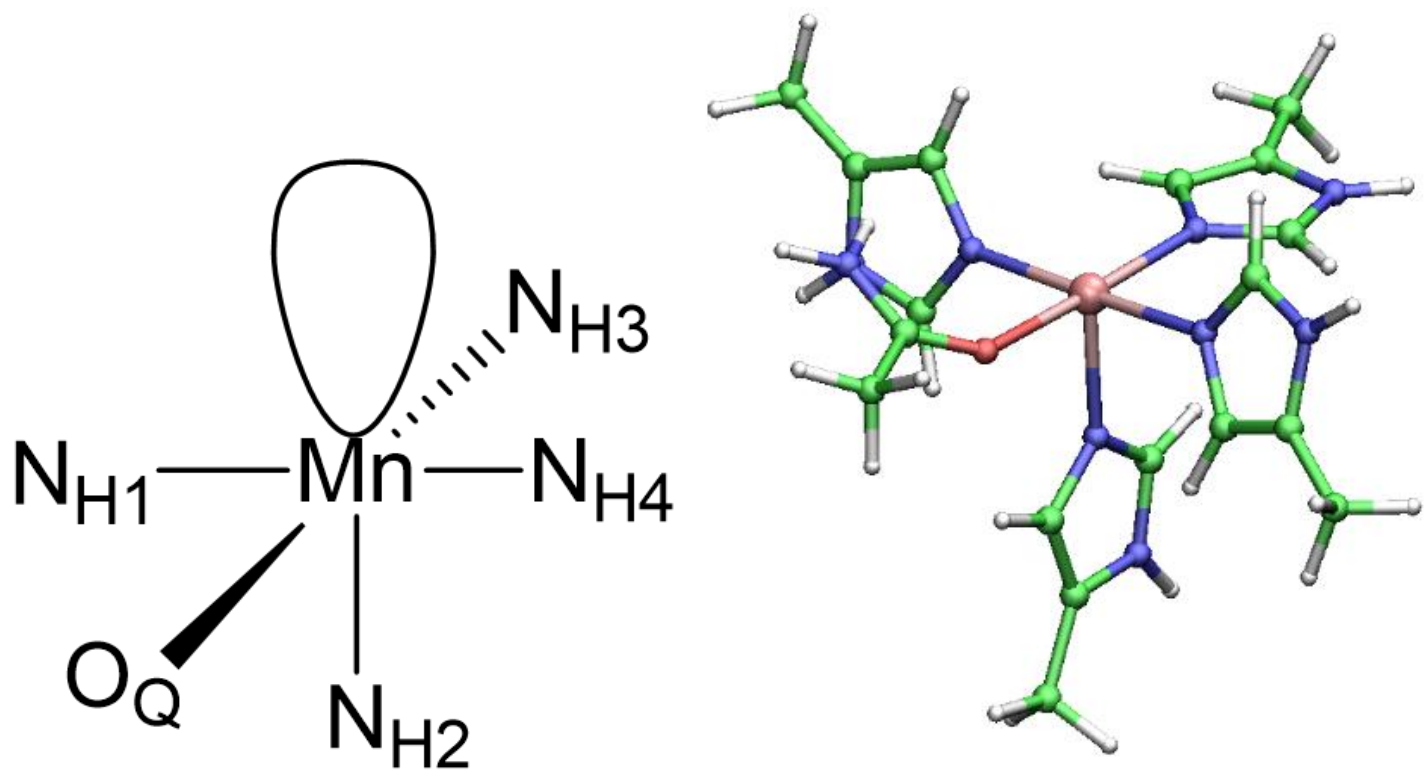

Figure S2. Optimized structure of **Mn-A** model with Mn(III), including first coordination sphere of the cation (Q=Q59; H1=H96; H2=H96; H3=H53; H4=H55).

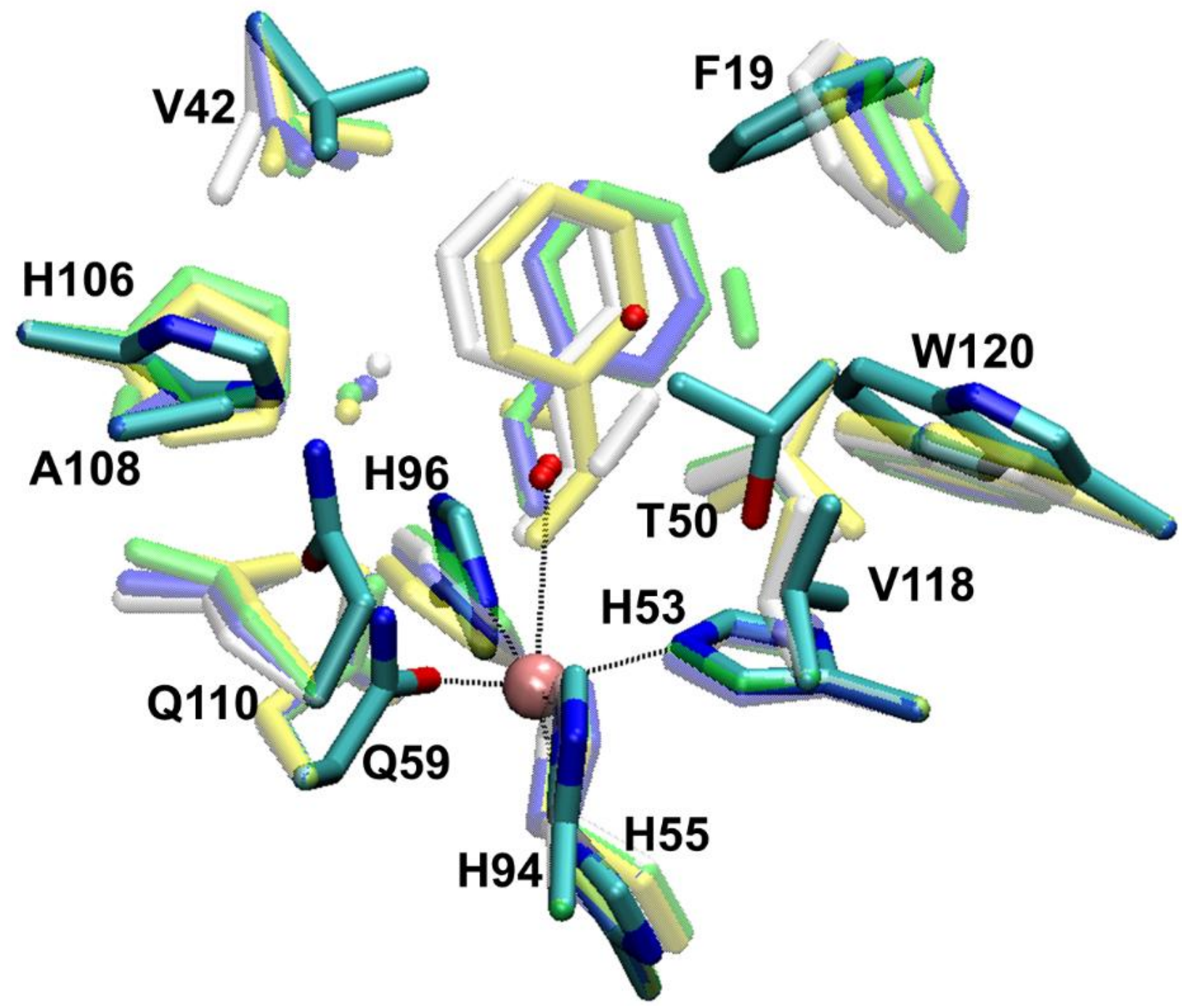

Figure S3. Superposition of crystallographic structure and optimized ESs (transparent representation).

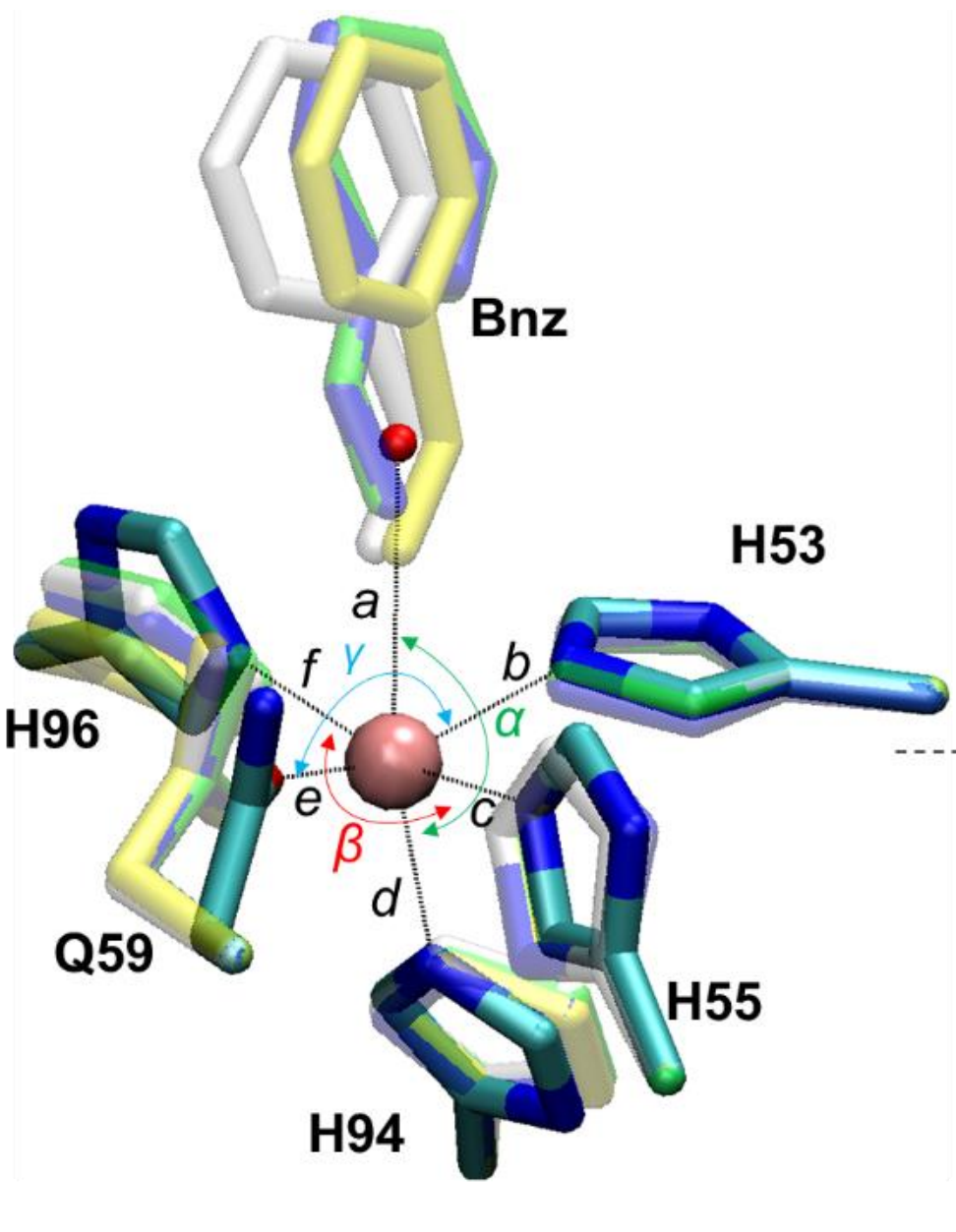

	4BIF	ES-$S^{Q59/V42}$	ES-$S^{T50/V118}$	ES-$R^{T50/V42}$	ES-$R^{Q59/V118}$
a	3.39	3.06	2.48	2.53	3.01
b	2.28	2.18	2.22	2.19	2.19
c	2.47	2.25	2.24	2.23	2.26
d	2.40	2.24	2.31	2.29	2.25
e	1.64	2.15	2.17	2.22	2.14
f	2.41	2.18	2.20	2.18	2.19
α	158.5	164.8	165.0	163.7	165.3
β	163.6	166.3	168.6	166.9	167.3
γ	166.9	159.0	167.2	163.9	160.3

Figure S4. Comparison of relevant geometric parameters of Mn(II) coordination sphere of crystallographic structure and optimized ESs.

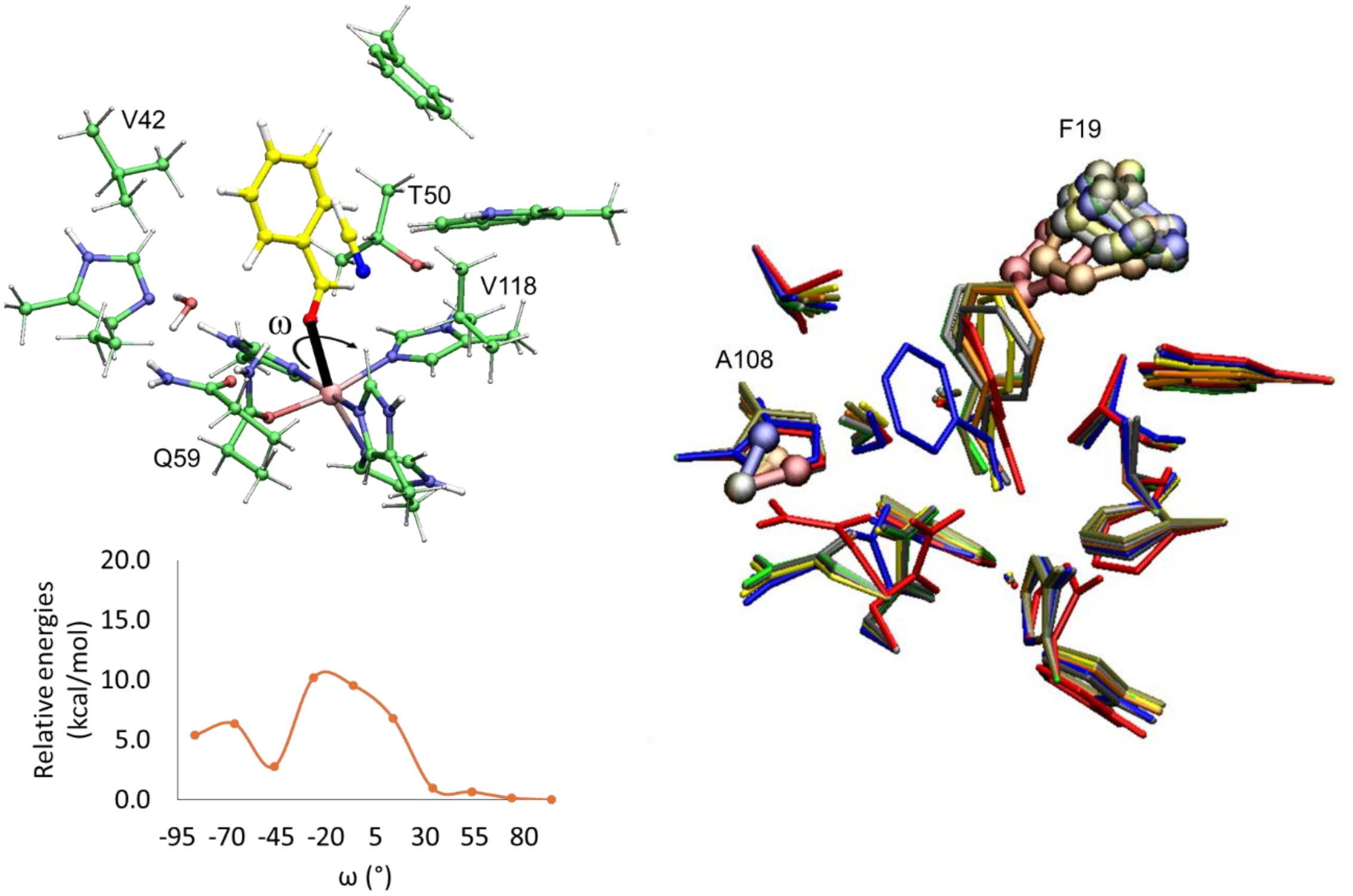

Figure S5. On the left, scanned dihedral angle for the study of **Bnz** flexibility in *Gt*HNL active site and relative relaxed energy profile. On the right, superposition of all the obtained structures.

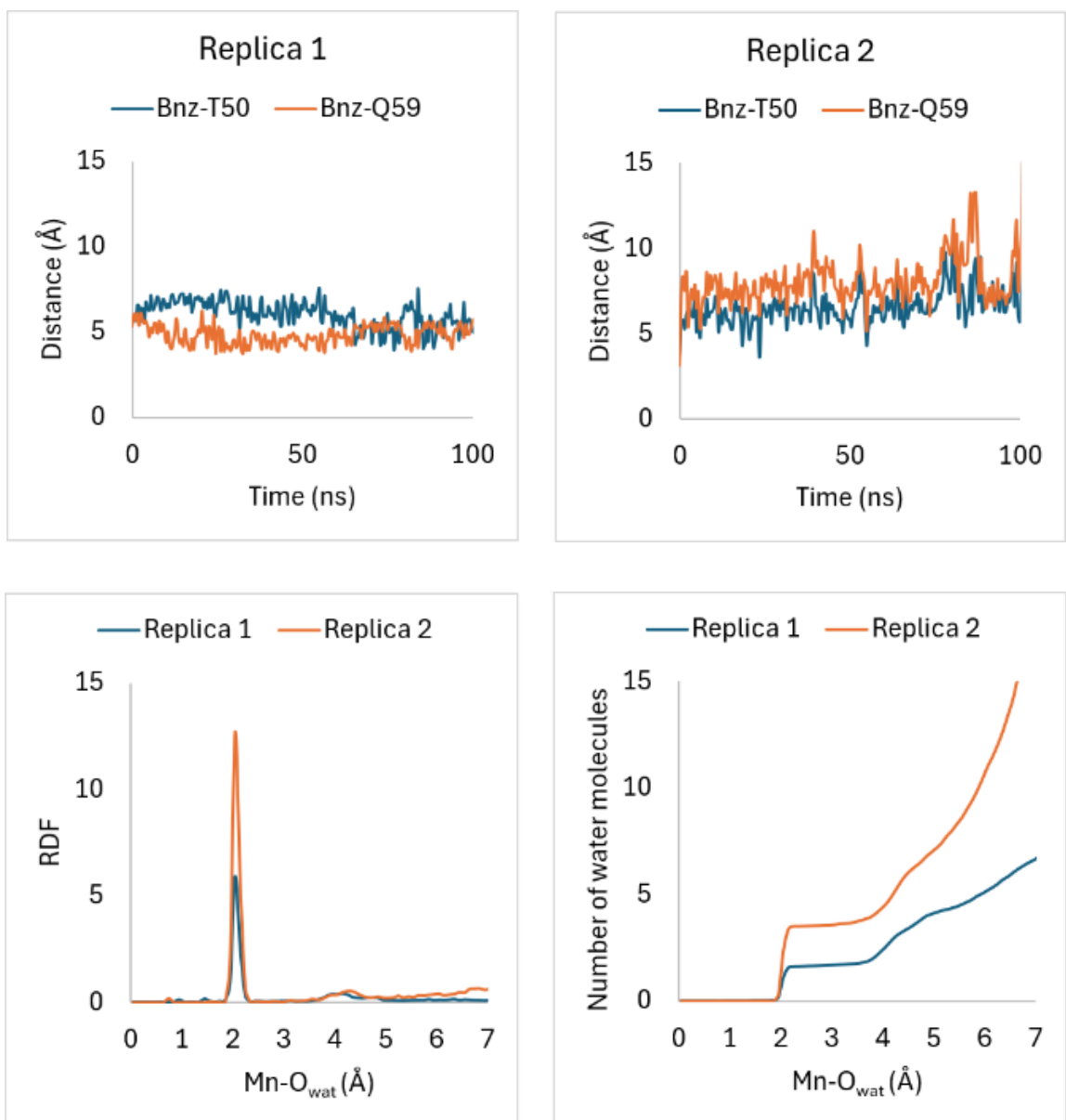

Figure S5. Results from MD simulation on 2x100 ns of *Gt*HNL:Bnz system. Technical details on simulations are provided below.

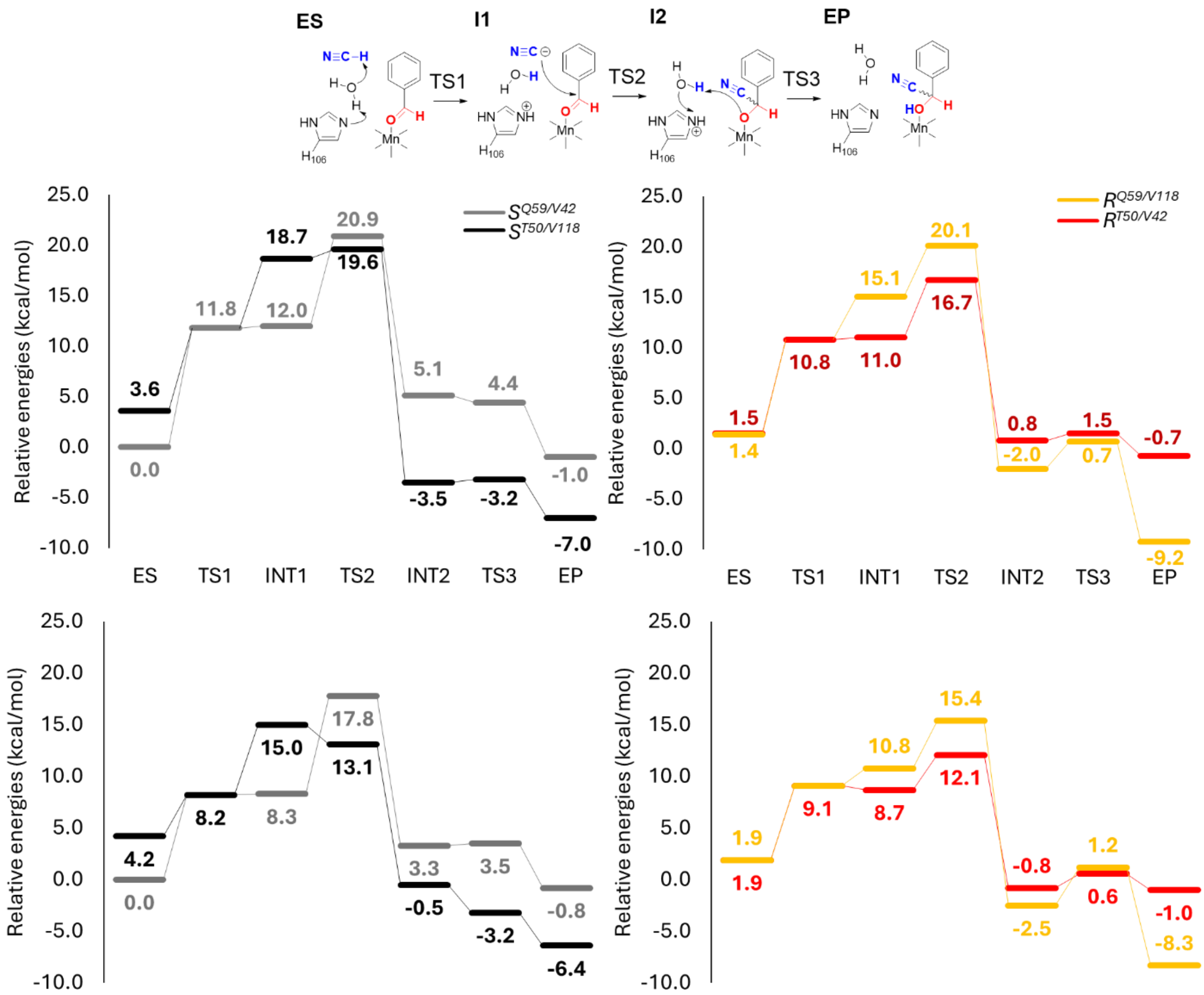

Figure S7. Energy profiles calculated for the formation of mandelonitrile, in ε=4.0 and ε=78.3 (top and bottom respectively).

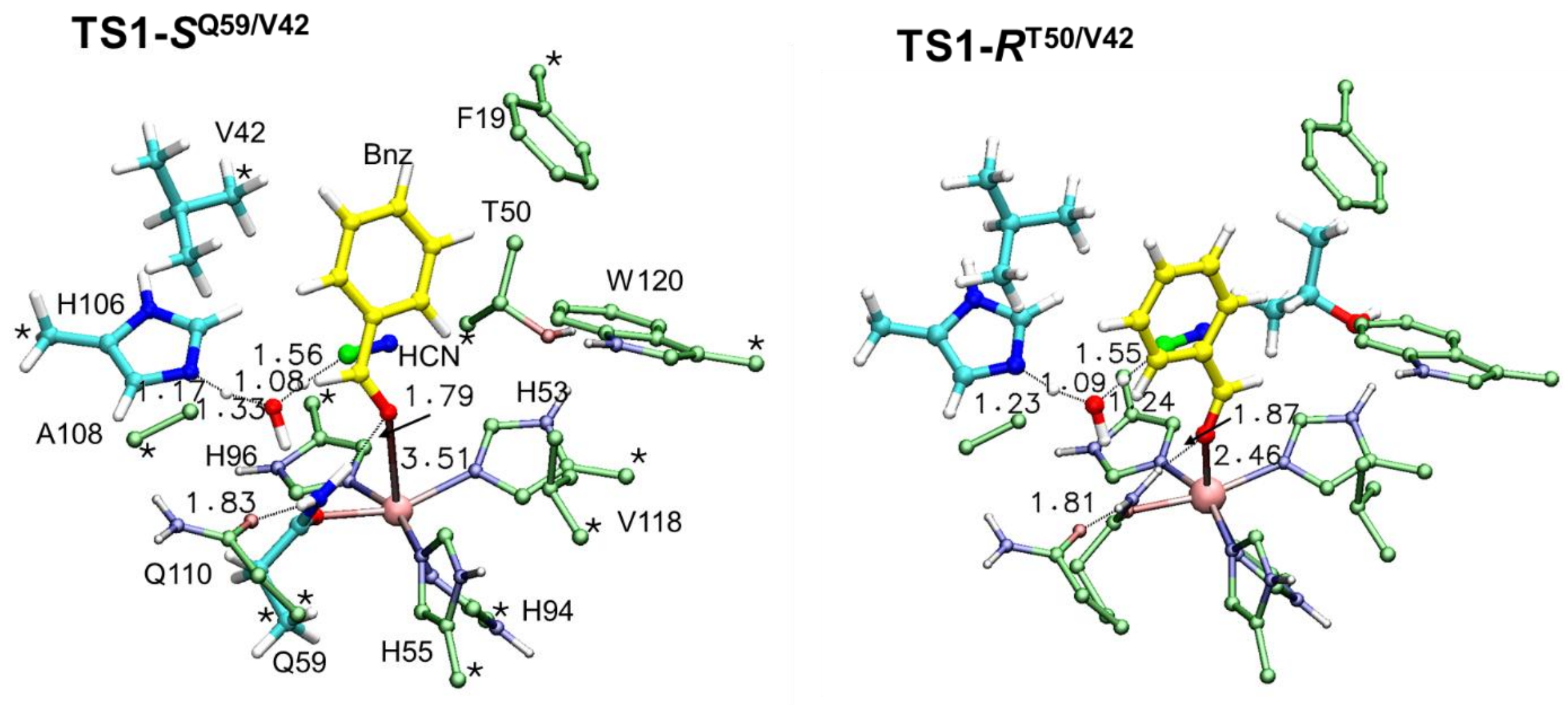

Figure S8. Optimized structures of lowest-energy transition states that lead to the enantiomers TS1-$S^{Q59/V42}$ and TS1-$R^{T50/V42}$. Selected distances are given in Å. For clarity, most of the hydrogens are omitted. Atoms kept fixed during the geometry optimizations are labeled with asterisk.

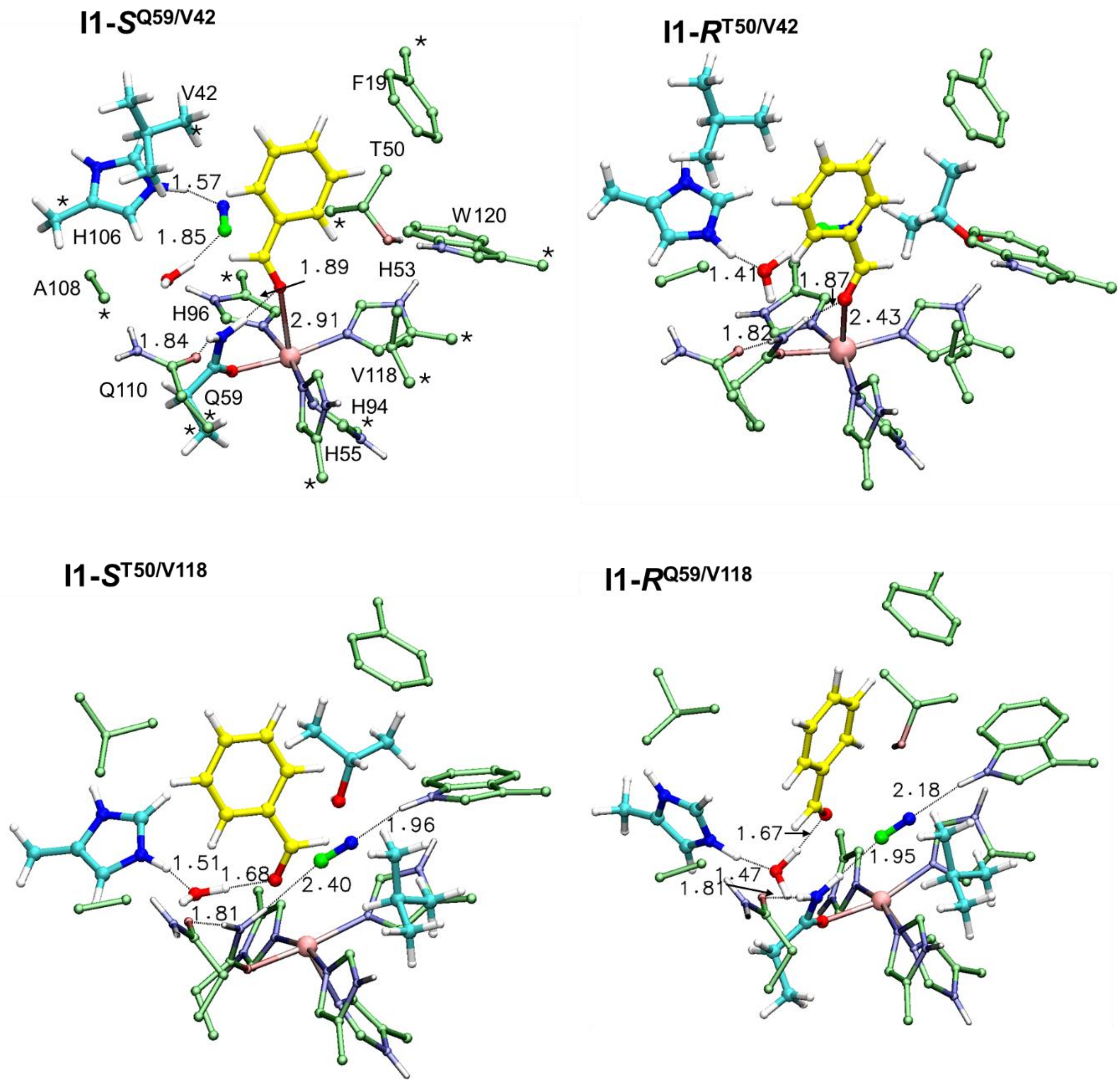

Figure S9. Optimized structures of **I1** intermediates that lead to the enantiomers *S* and *R*. Selected distances are given in Å. For clarity, most of the hydrogens are omitted. Atoms kept fixed during the geometry optimizations are labeled with asterisk.

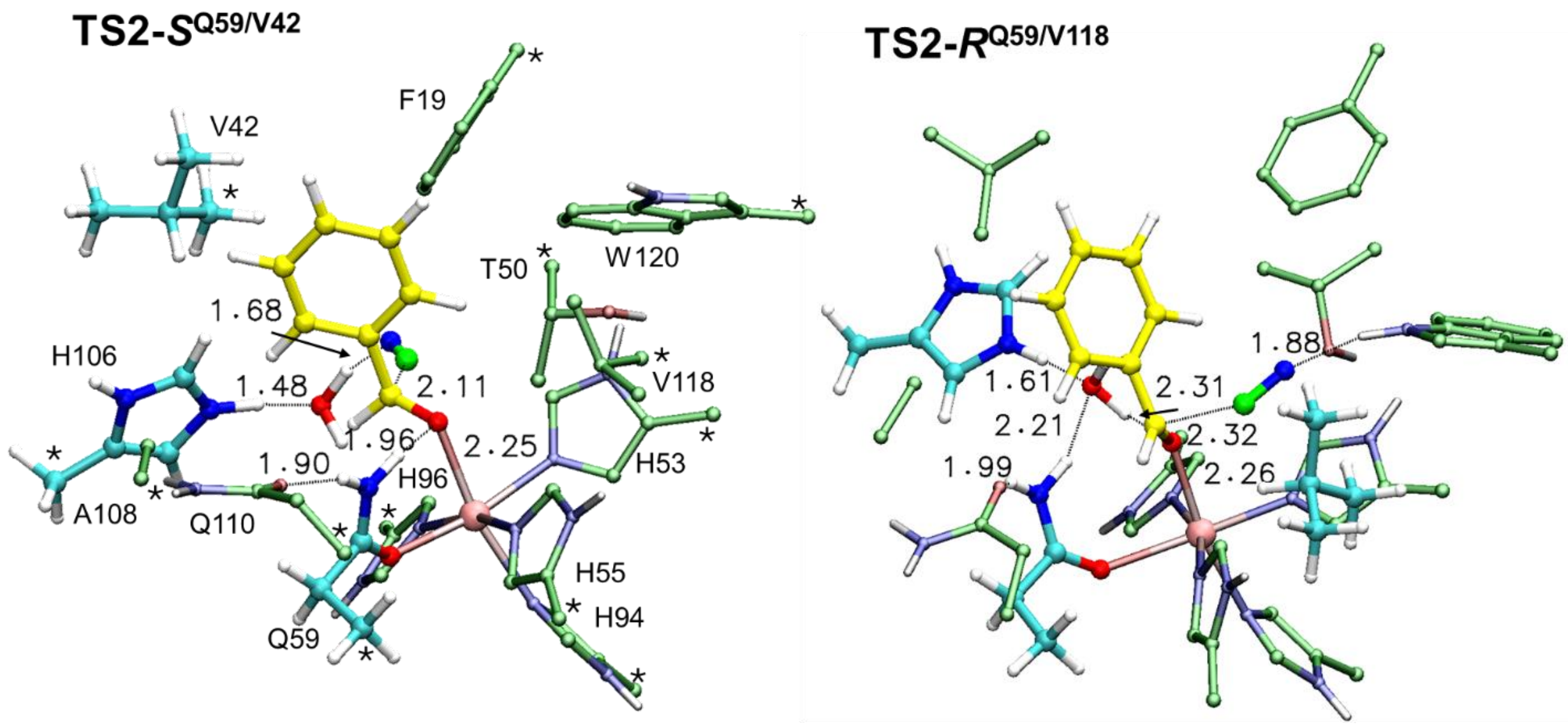

Figure S10. Optimized structures of **TS2-*S*$^{Q59/V42}$ and TS2-*R*$^{Q59/V118}$.** that lead to the enantiomers *S* and *R*. Selected distances are given in Å. For clarity, most of the hydrogens are omitted. Atoms kept fixed during the geometry optimizations are labeled with asterisk.

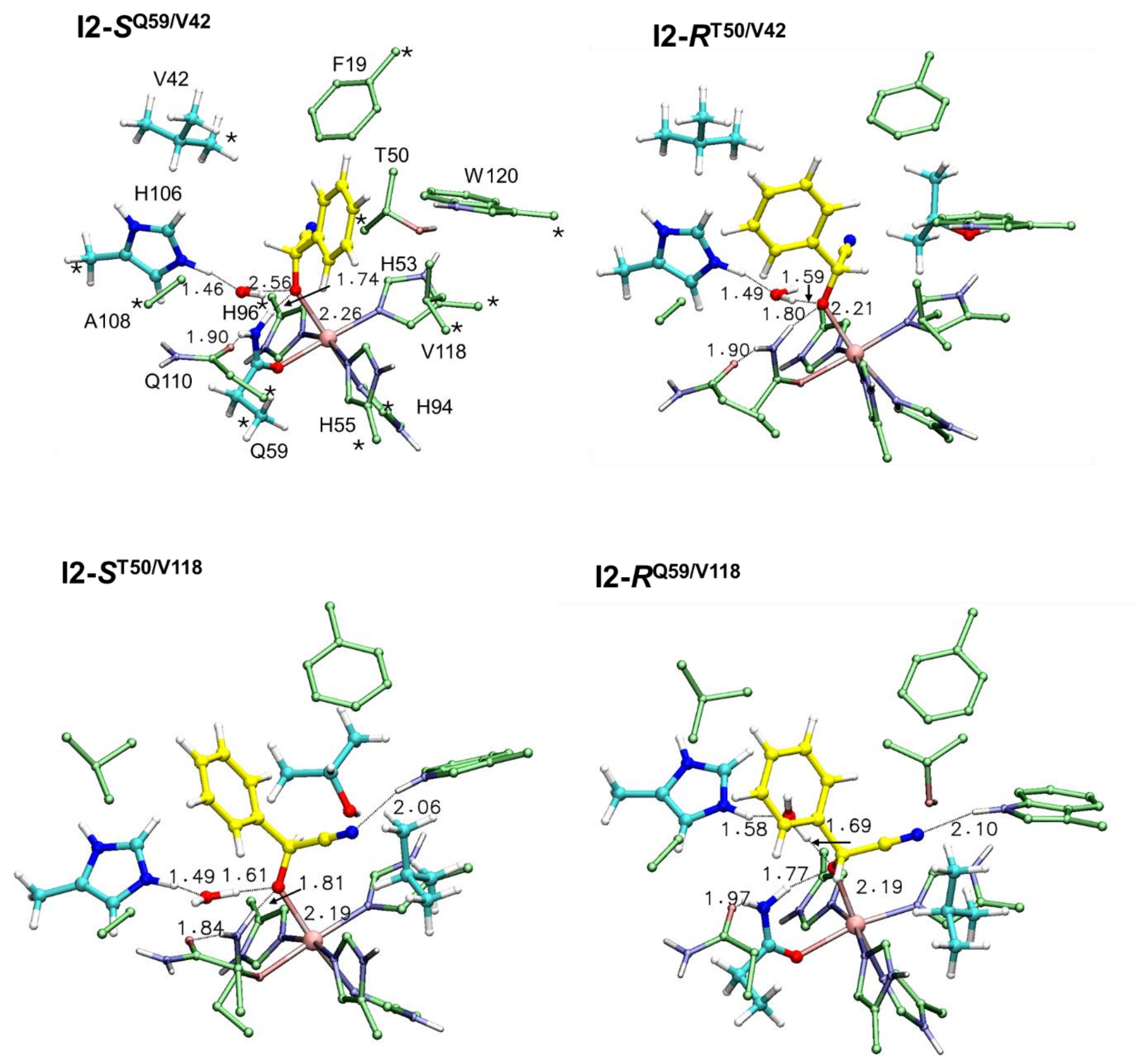

Figure S11. Optimized structures of **I2** intermediates that lead to the enantiomers *S* and *R*. Selected distances are given in Å. For clarity, most of the hydrogens are omitted. Atoms kept fixed during the geometry optimizations are labeled with asterisk.

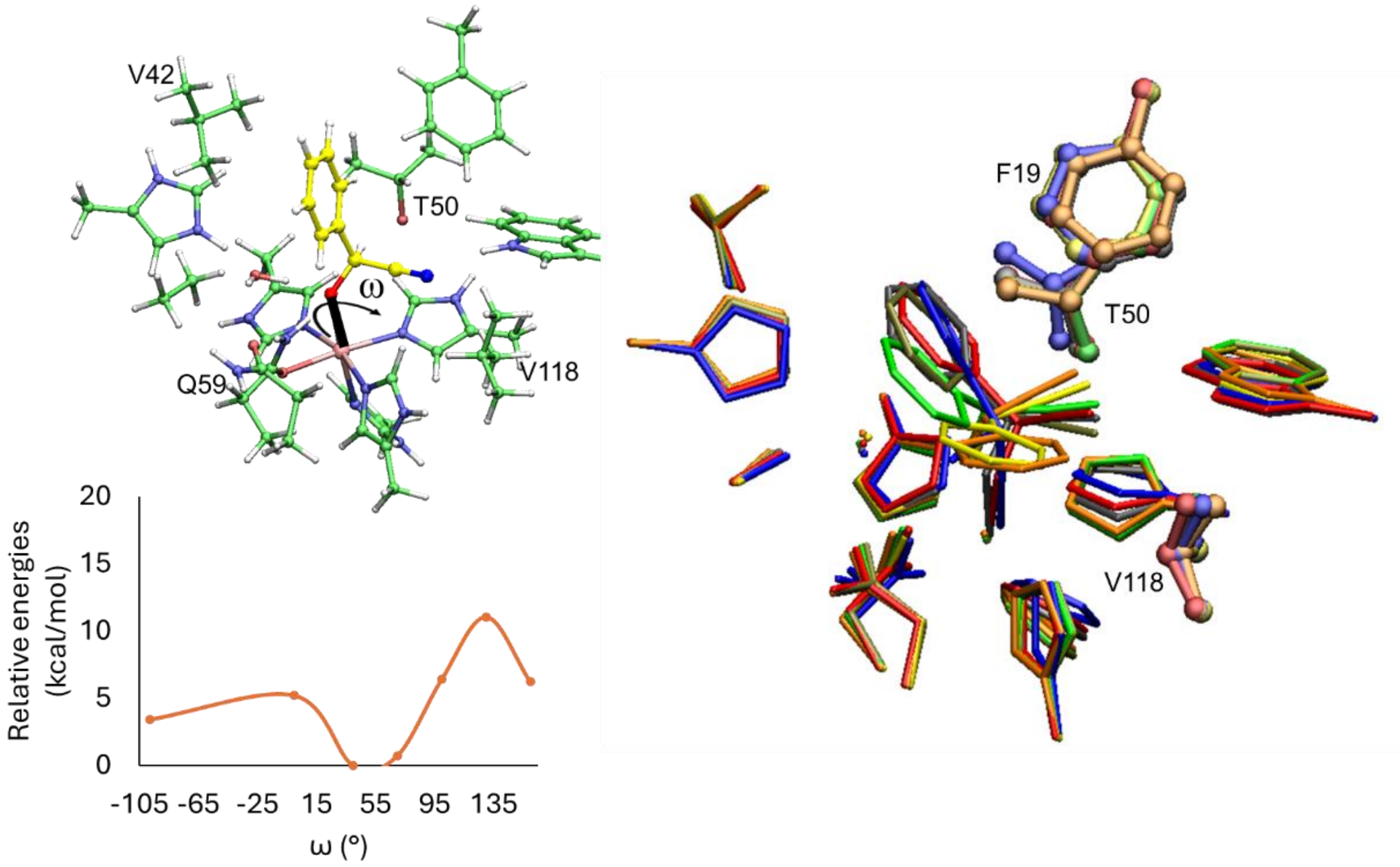

Figure S12. On the left, scanned dihedral angle for the study of deprotonated **Mnd** flexibility in *Gt*HNL active site and relative relaxed energy profile. On the right, superposition of all the obtained structures.

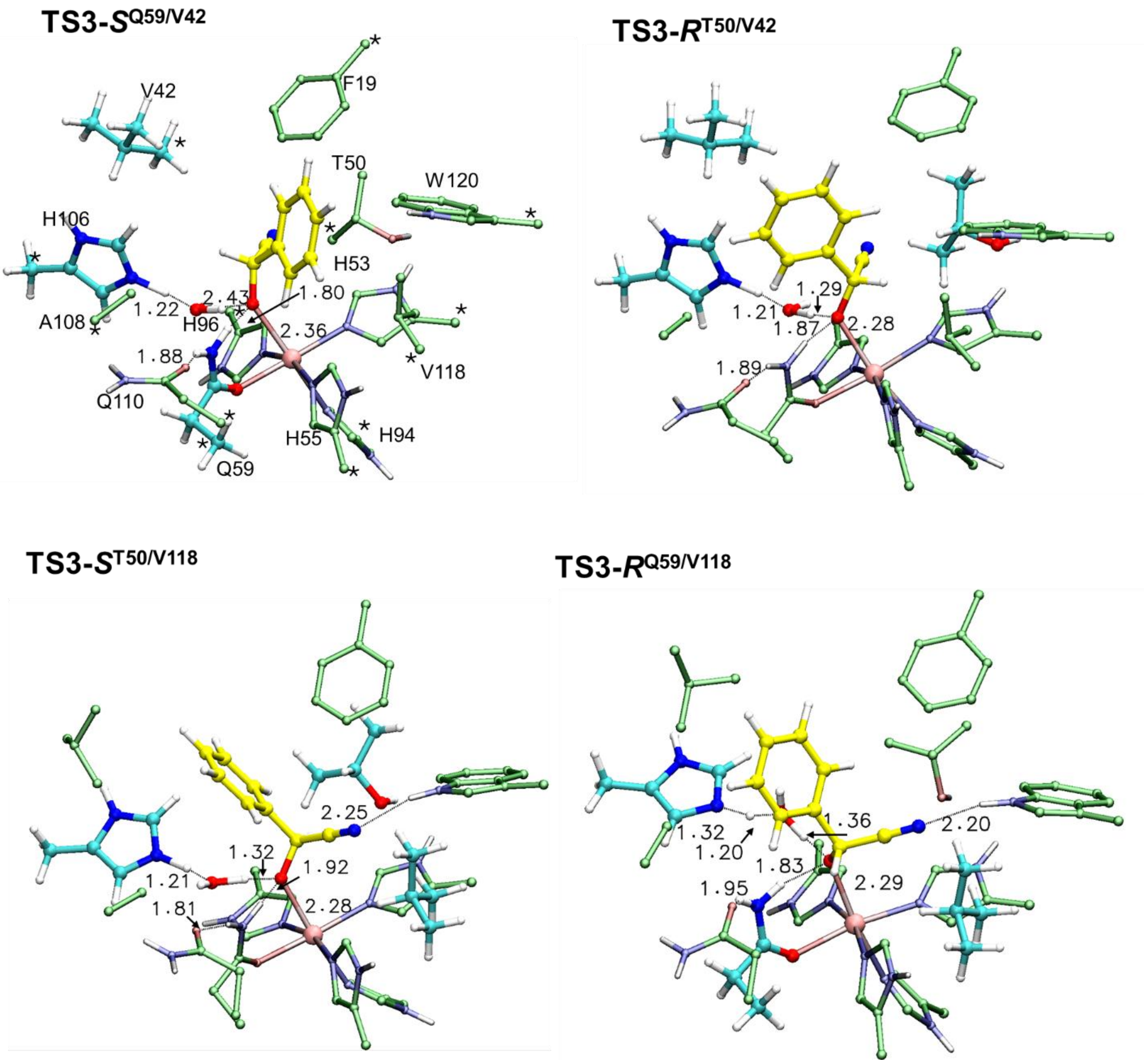

Figure S13. Optimized structures of **TS3** transition states that lead to the enantiomers *S* and *R*. Reported distances are given in Å. For clarity, most of the hydrogens are omitted. Atoms kept fixed during the geometry optimizations are labeled with asterisks.

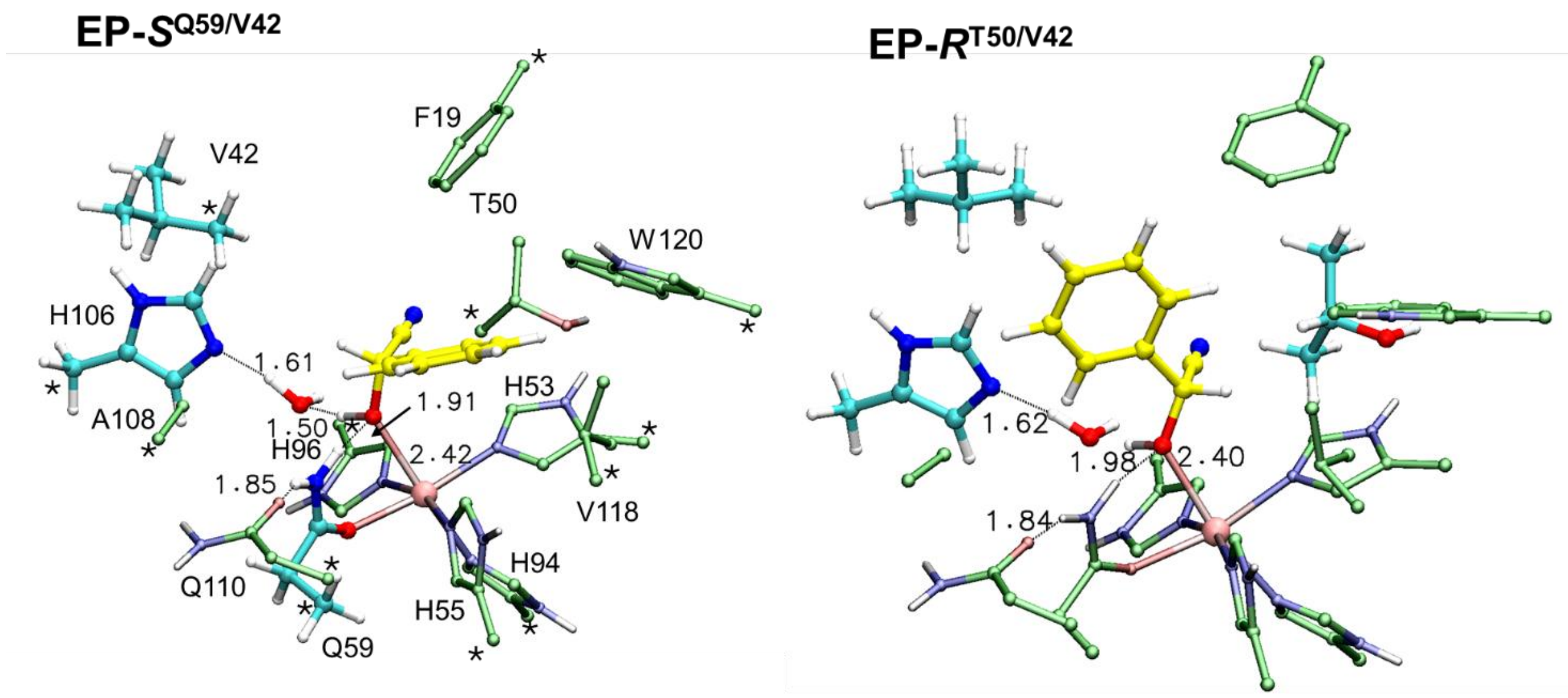

Figure S14. Optimized structures of **EP-$S^{Q59/V42}$** and **EP-$R^{Q59/V118}$**. Selected distances are given in Å. For clarity, most of the hydrogens are omitted. Atoms kept fixed during geometry optimizations are labeled with asterisks.

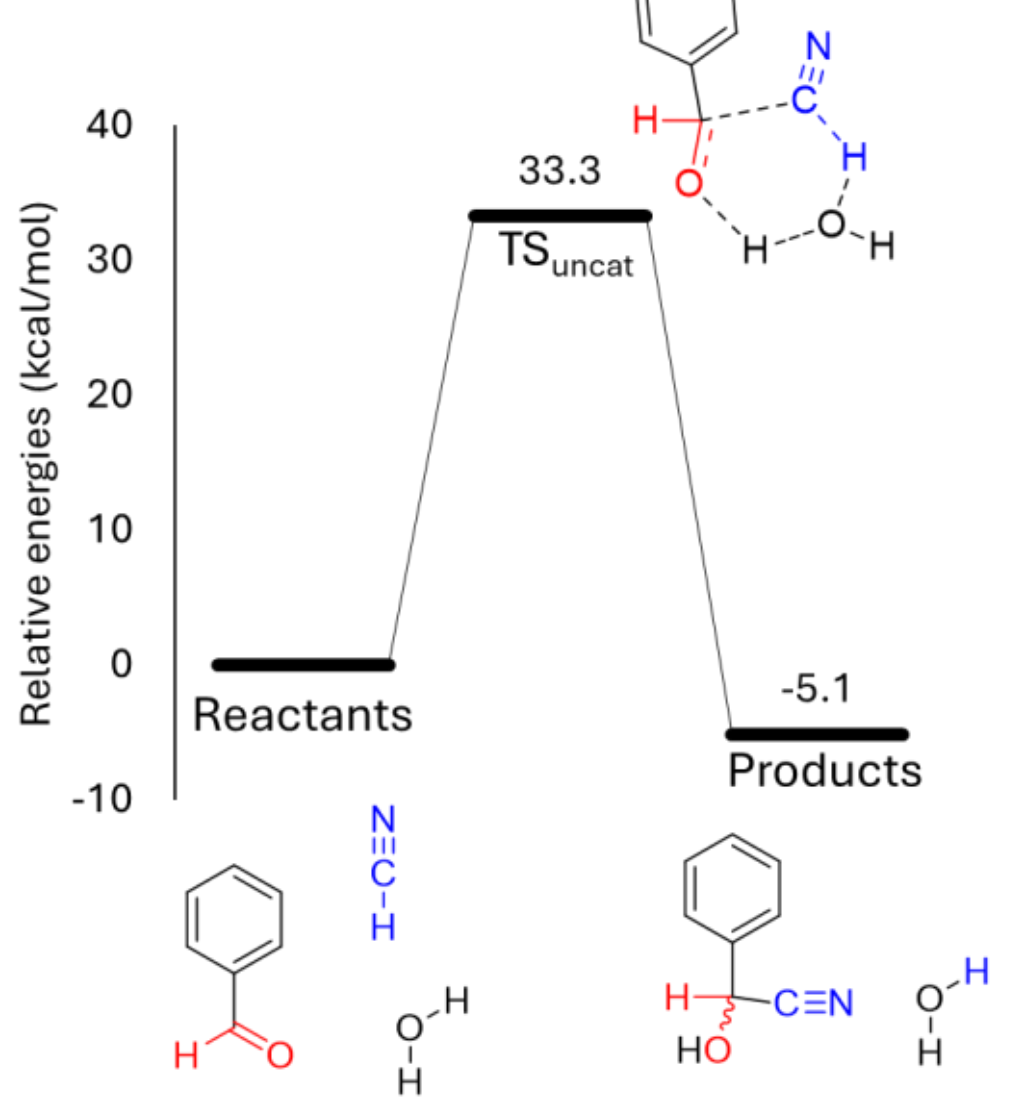

Figure S15. Calculated energy profile for uncatalyzed formation of mandelonitrile.

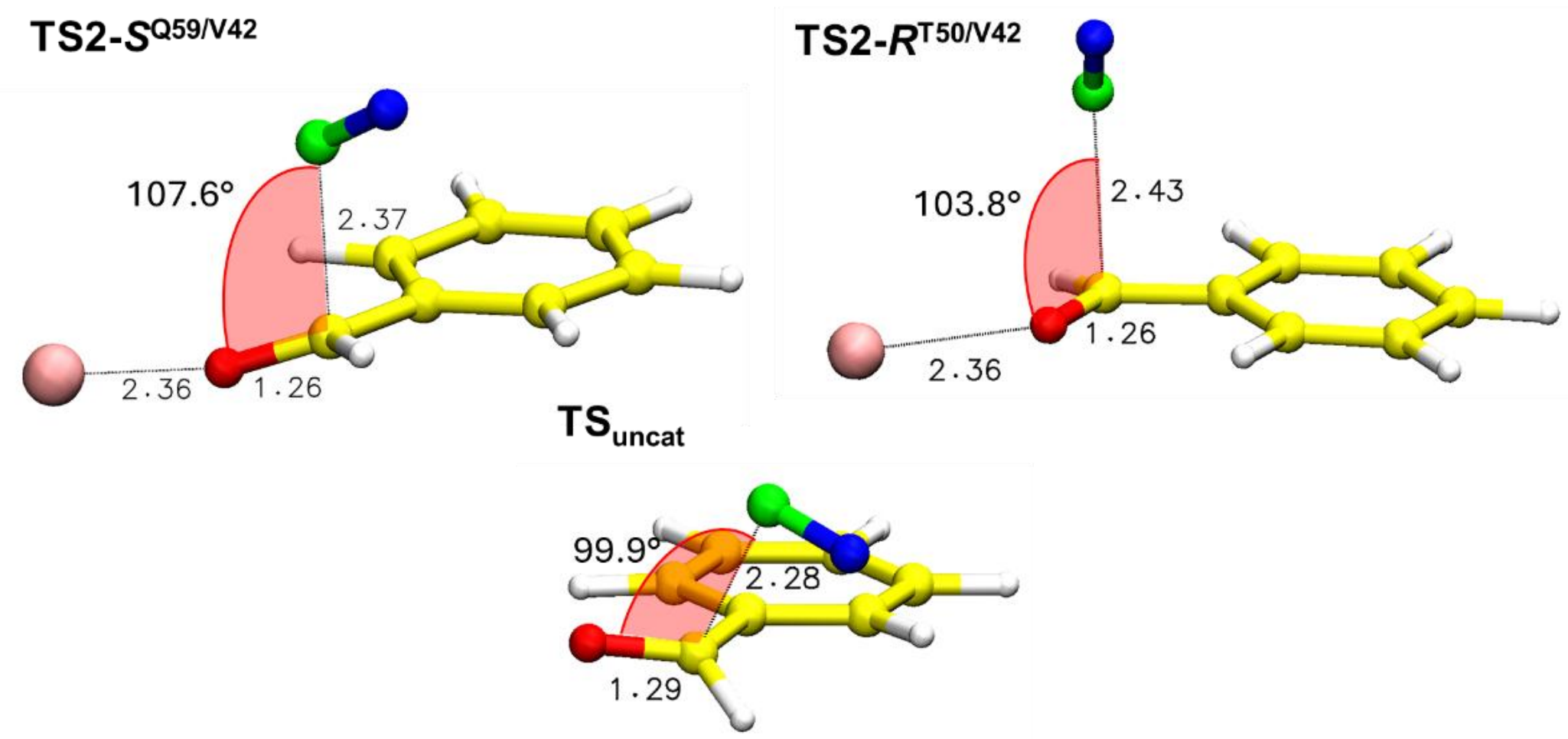

Figure S16. Focus on geometrical parameters characterizing the lowest-energy TS2-$S^{Q59/V42}$ and TS2-$R^{T50/V42}$ and the transition state of the uncatalyzed reaction (TS_{uncat}).

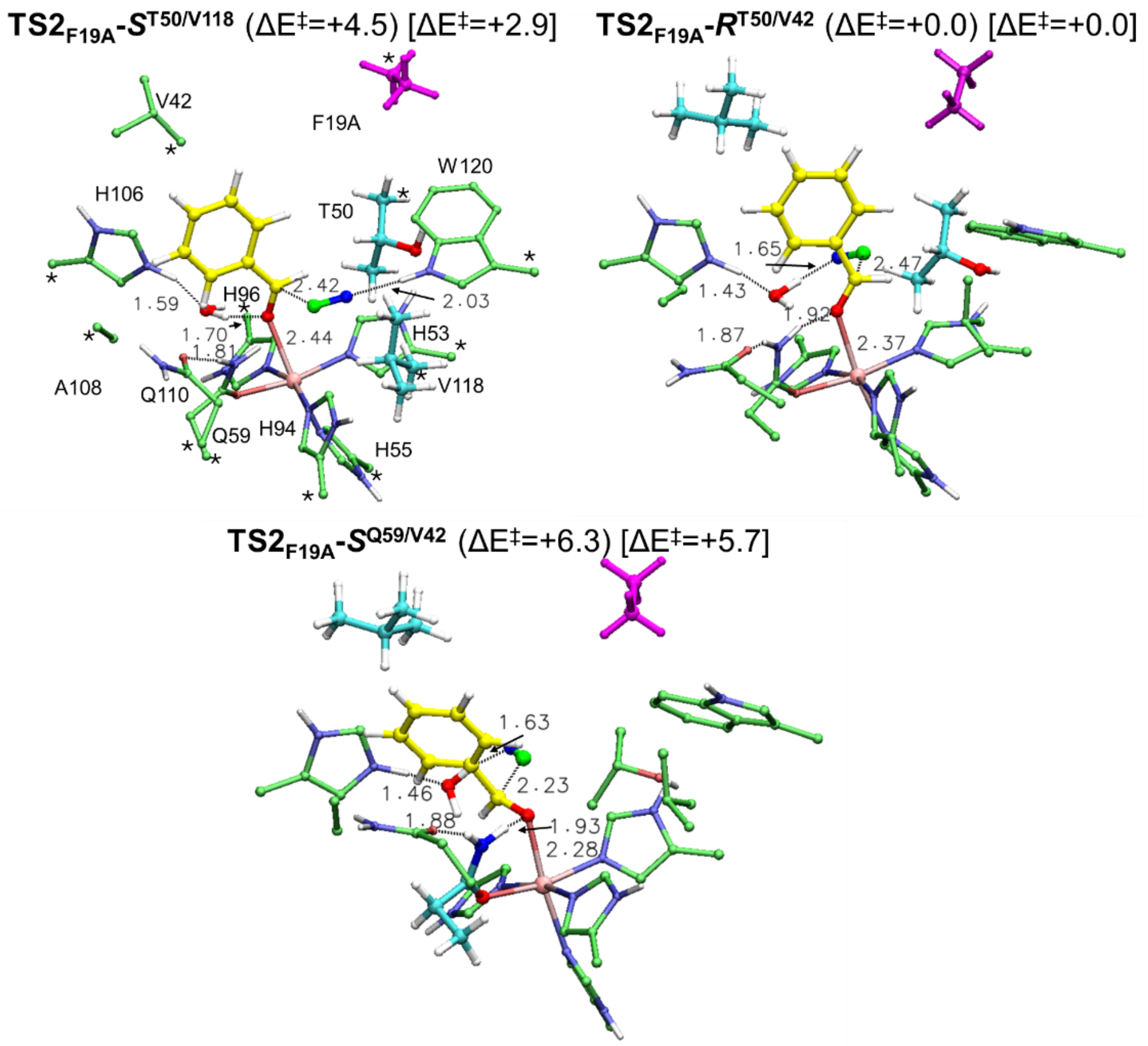

Figure S17. Optimized structures of **TS2** with F19A mutations that lead to the enantiomers *S* and *R*. Reported distances are given in Å. Relative energies in round and square parenthesis (for protein and water environment respectively) are indicated in kcal mol^{-1}. For clarity, most of the hydrogens are omitted. Atoms kept fixed during the geometry optimizations are labeled with asterisks.

TS2$_{\mathbf{W120A}}$-***S***$^{\mathbf{T50/V118}}$ ($\Delta E^{\ddagger}$=+16.4) [$\Delta E^{\ddagger}$=+10.3] **TS2**$_{\mathbf{W120A}}$-***R***$^{\mathbf{T50/V42}}$ ($\Delta E^{\ddagger}$=+0.0) [$\Delta E^{\ddagger}$=+0.0]

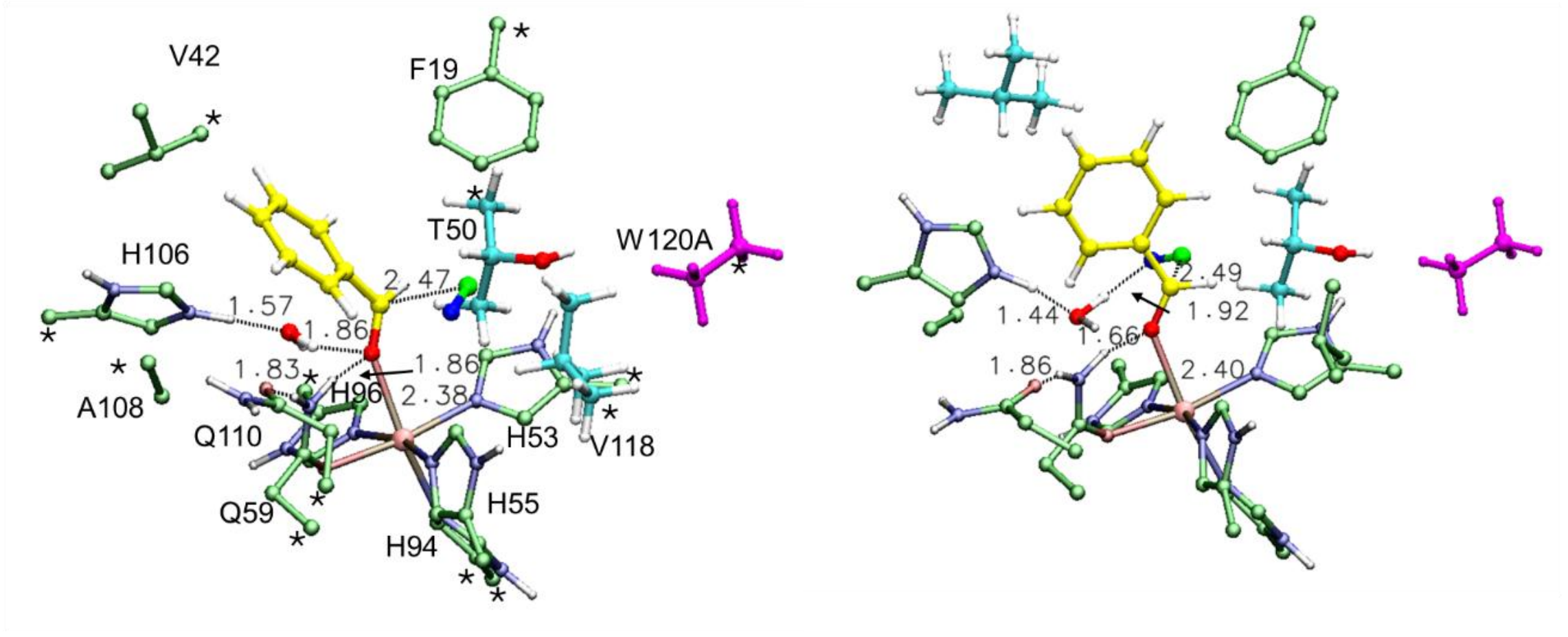

TS2$_{\mathbf{W120A}}$-***S***$^{\mathbf{Q59/V42}}$ ($\Delta E^{\ddagger}$=+2.2) [$\Delta E^{\ddagger}$=+1.9]

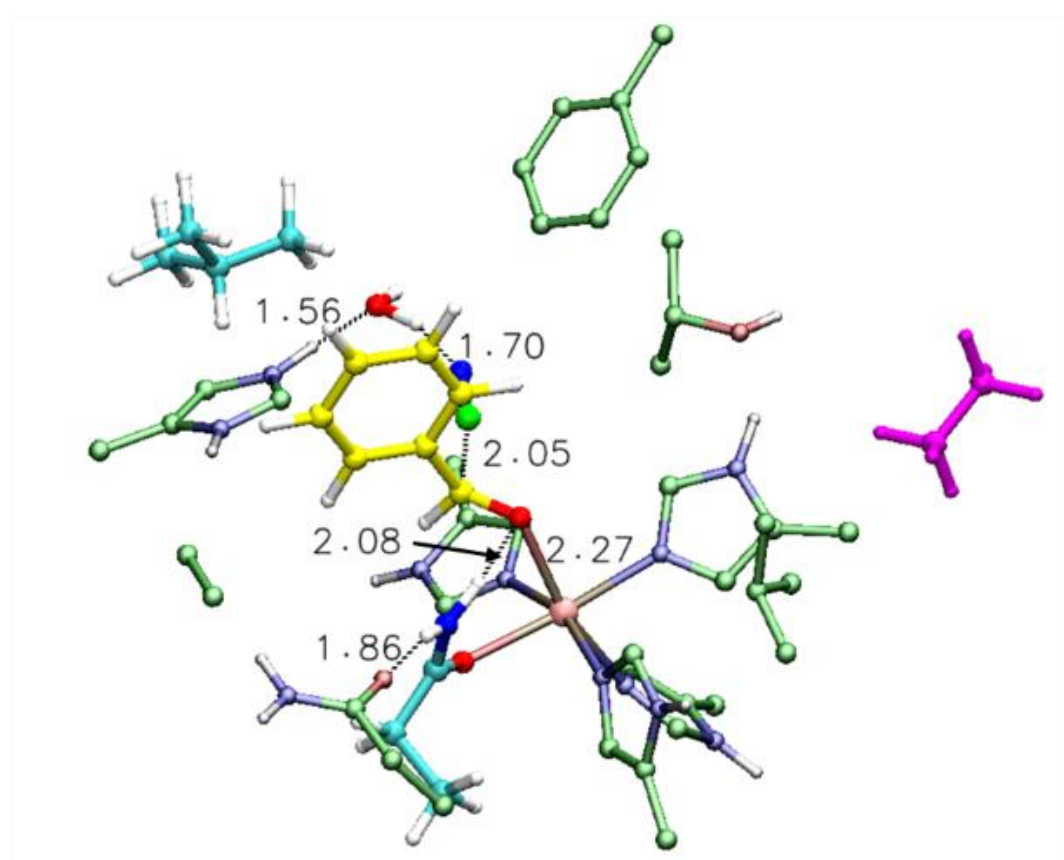

Figure S18. Optimized structures of **TS2** with W120A mutations that lead to the enantiomers *S* and *R*. Reported distances are given in Å. Relative energies in round and square parenthesis (for protein and water environment respectively) are indicated in kcal mol^{-1}. For clarity, most of the hydrogens are omitted. Atoms kept fixed during geometry optimizations are labeled with asterisks.

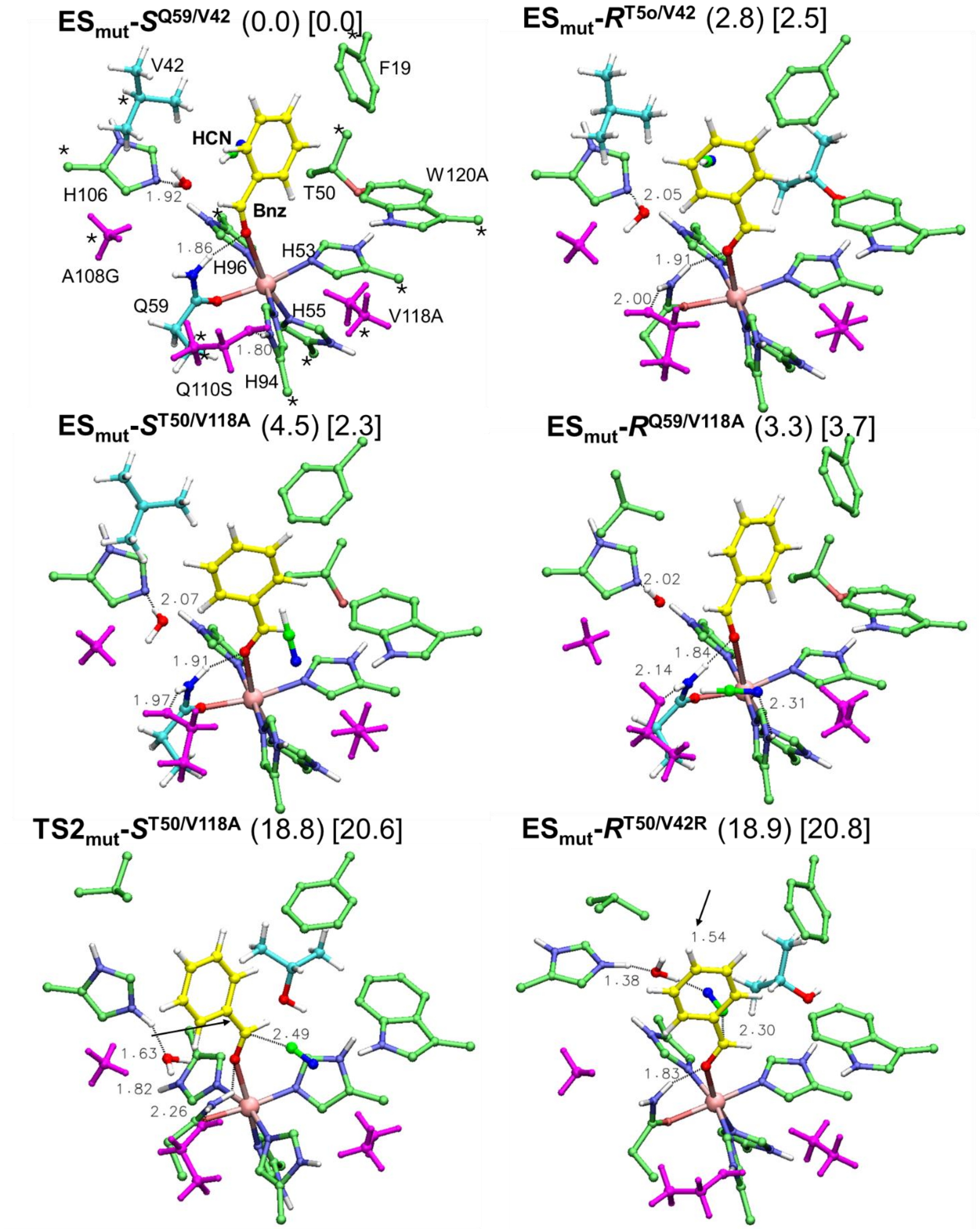

Figure S19. Optimized structures of **ES** complexes and **TS2** with A108G, Q110S and V118A mutations that lead to the enantiomers *S* and *R*. Reported distances are given in Å. Relative energies in parenthesis are indicated in kcal mol^{-1}. For clarity, most of the hydrogens are omitted. Atoms kept fixed during geometry optimizations are labeled with asterisks.

Table S1. Spin densities on Mn^{2+}, calculated for all the optimized structures.

	ρ		ρ
**ES-$S^{Q59/V42}$**	5.067	**ES-$R^{T50/V42}$**	4.996
**TS1-$S^{Q59/V42}$**	5.013	**TS1- $R^{T50/V42}$**	5.004
**I1-$S^{Q59/V42}$**	5.021	**I1-$R^{T50/V42}$**	5.001
**TS2-$S^{Q59/V42}$**	4.897	**TS2-$R^{T50/V42}$**	5.002
**I2-$S^{Q59/V42}$**	5.022	**I2-$R^{T50/V42}$**	4.946
**TS3-$S^{Q59/V42}$**	5.027	**TS3-$R^{T50/V42}$**	4.967
**EP-$S^{Q59/V42}$**	5.062	**EP-$R^{T50/V42}$**	5.020
**ES-$S^{T50/V118}$**	4.988	**ES-$R^{Q59/V118}$**	5.077
**I1-$S^{T50/V118}$**	5.014	**I1-$R^{Q59/V118}$**	4.976
**TS2-$S^{T50/V118}$**	5.011	**TS2-$R^{Q59/V118}$**	5.001
**I2-$S^{T50/V118}$**	4.986	**I2-$R^{Q59/V118}$**	5.008
**TS3-$S^{T50/V118}$**	4.990	**TS3-$R^{Q59/V118}$**	4.907
**EP-$S^{T50/V118}$**	4.985	**EP-$R^{Q59/V118}$**	4.995

Table S2. Fukui parameter f^+ calculated for **Mn-A** and **Mn-B** species (for clarity, results of hydrogen atoms were omitted).

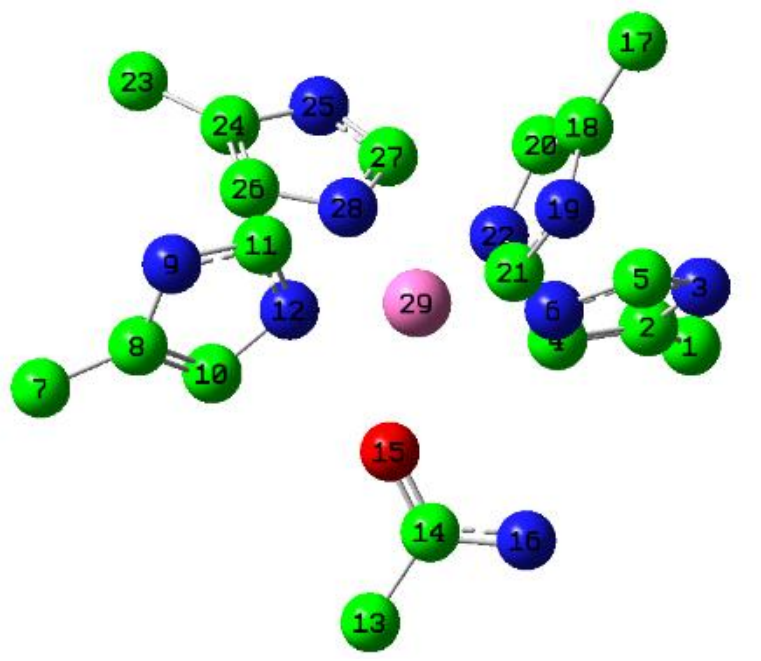

	**Mn-A**	**Mn-B**		**Mn-A**	**Mn-B**
**C1**	-845	40	**N16**	2730	2783
**C2**	3476	3258	**C17**	-812	-805
**N3**	1714	1910	**C18**	3294	3370
**C4**	-659	-660	**N19**	2203	1739
**C5**	3859	3370	**C20**	-672	-993
**N6**	-9486	-9585	**C21**	3955	4796
**C7**	-795	-810	**N22**	-10198	-8236
**C8**	3514	3420	**C23**	-872	-884
**N9**	1537	1625	**C24**	3713	3631
**C11**	2141	2762	**N25**	1893	2071
**N12**	-9052	-9900	**C26**	-130	234
**C13**	-340	-262	**C27**	3678	4889
**C14**	5631	5575	**N28**	-11882	-12153
**O15**	-8692	-7741	**Mn29**	76054	72220

Table S3. Hirshfield-CM5 charges calculated for optimized ES complexes (Mulliken population in parenthesis).

	$N\varepsilon_{H106}$	O_{Bnz}	O_{w1}	C_{HCN}	C_{Bnz}
**ES-*S***$^{Q59/V42}$	-0.36 (-0.72)	-0.28 (-0.42)	-0.63 (-0.44)	0.12 (-0.25)	0.13 (0.26)
**ES-*R***$^{T50/V42}$	-0.36 (-0.63)	-0.27 (-0.21)	-0.61 (-0.44)	0.11 (-0.27)	0.13 (0.23)
**ES-*S***$^{T50/V118}$	-0.36 (-0.65)	-0.25 (-0.13)	-0.62 (-0.37)	0.14 (-0.20)	0.14 (0.27)
**ES-*R***$^{Q59/V118}$	-0.36 (-0.65)	-0.28 (-0.41)	-0.62 (-0.40)	0.14 (-0.20)	0.14 (0.27)
**I1-*S***$^{Q59/V42}$	-0.01 (-0.31)	-0.21 (-0.31)	-0.22 (-0.47)	-0.15 (-0.58)	0.14 (0.09)
**I1-*R***$^{T50/V42}$	-0.03 (-0.24)	-0.18 (-0.40)	-0.24 (-0.39)	-0.19 (-0.73)	0.16 (0.19)
**I1-*S***$^{T50/V118}$	-0.02 (-0.28)	-0.19 (-0.41)	-0.23 (-0.54)	-0.17 (-0.89)	0.15 (0.14)
**I1-*R***$^{Q59/V118}$	-0.02 (-0.32)	-0.20 (-0.31)	-0.25 (-0.46)	-0.19 (-0.60)	0.12 (-0.01)

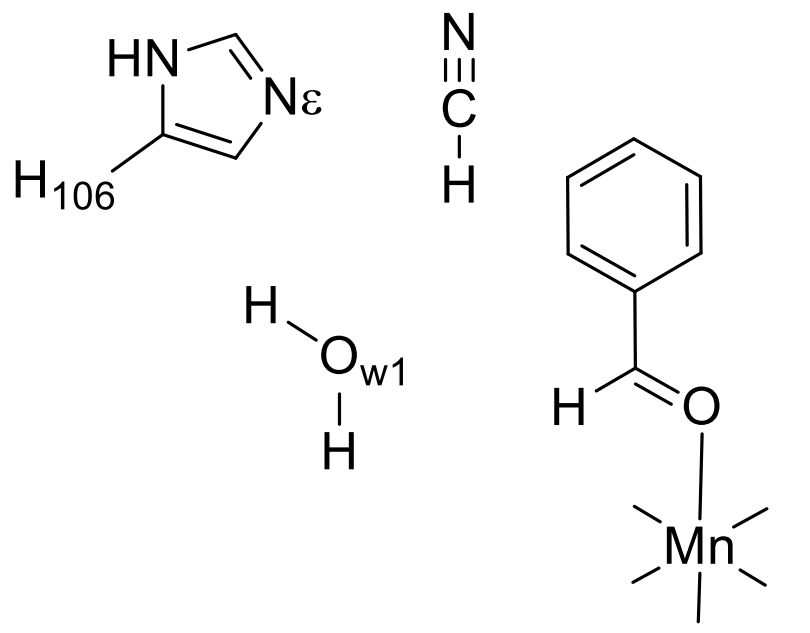

Table S4. Affinities for **Bnz** and **HCN** substrates in **ES-*S***$^{Q59/V42}$, **TS2-*R***$^{T50/V42}$ and **TS2-*S***$^{T50/V118}$, calculated via counterpoise method (CP) at SDD/B3LYP-D3/6-31G(d,p) level of theory, for full model and fragments, *i.e.* full model without F19/Q59/V118/W120. Values are in kcal/mol.

ΔΔE = $CP_{Full\ model}$ - $CP_{fragment}$

	**$CP_{Full\ model}$**	**CP_{F19}**	**ΔΔE**	**CP_{Q59}**	**ΔΔE**	**CP_{V118}**	**ΔΔE**	**CP_{W120}**	**ΔΔE**
**ES-*S***$^{Q59/V42}$	-36.6	-32.5	4.1	-35.2	1.4	-36.6	0.0	-35.4	1.2
**TS2-*R***$^{T50/V42}$	-160.6	-158.3	2.3	-163.1	-2.5	-160.5	0.8	-158.7	11.5
**TS2-*S***$^{T50/V118}$	-195.6	-193.1	2.5	-188.3	7.3	-194.8	0.2	-184.1	1.9

Table S5. Relevant geometric parameters (in Å) of optimized intermediates and transition states. Mean value (d) and standard deviation (σ) are also reported.

	Mn-O_{Bnz}	Mn-N_{H55}	Mn-N_{H94}	Mn-O_{Q59}	Mn-N_{H96}	Mn-N_{H53}	C_{Bnz}-C_{HCN}
**ES-*S*$^{Q59/V42}$**	3.06	2.24	2.25	2.15	2.18	2.18	4.16
**TS1-*S*$^{Q59/V42}$**	3.51	2.27	2.23	2.23	2.20	2.16	3.46
**I1-*S*$^{Q59/V42}$**	2.91	2.26	2.24	2.18	2.18	2.18	2.97
**TS2-*S*$^{Q59/V42}$**	2.25	2.38	2.28	2.22	2.25	2.19	2.11
**I2-*S*$^{Q59/V42}$**	2.26	2.50	2.29	2.22	2.24	2.18	1.49
**TS3-*S*$^{Q59/V42}$**	2.36	2.45	2.30	2.20	2.23	2.17	1.49
**EP-*S*$^{Q59/V42}$**	2.42	2.36	2.26	2.20	2.23	2.20	1.48
**d**	2.68	2.35	2.26	2.20	2.21	2.18	2.45
**σ**	0.49	0.10	0.03	0.03	0.03	0.01	1.09
**ES-*R*$^{T50/V42}$**	2.53	2.29	2.23	2.22	2.18	2.19	3.02
**TS1- *R*$^{T50/V42}$**	2.46	2.37	2.24	2.29	2.21	2.18	2.89
**I1-*R*$^{T50/V42}$**	2.43	2.37	2.24	2.28	2.21	2.17	2.93
**TS2-*R*$^{T50/V42}$**	2.36	2.34	2.27	2.24	2.20	2.19	2.43
**I2-*R*$^{T50/V42}$**	2.21	2.45	2.28	2.24	2.23	2.24	1.49
**TS3-*R*$^{T50/V42}$**	2.28	2.41	2.28	2.22	2.22	2.24	1.49
**EP-*R*$^{T50/V42}$**	2.40	2.32	2.27	2.20	2.22	2.22	1.48
**d**	2.38	2.37	2.26	2.24	2.21	2.20	2.25
**σ**	0.11	0.05	0.02	0.04	0.02	0.03	0.73
**ES-*S*$^{T50/V118}$**	2.48	2.31	2.24	2.17	2.20	2.22	3.47
**I1-*S*$^{T50/V118}$**	2.36	2.32	2.29	2.18	2.25	2.20	2.39
**TS2-*S*$^{T50/V118}$**	2.36	2.31	2.29	2.18	2.24	2.21	2.37
**I2-*S*$^{T50/V118}$**	2.19	2.42	2.27	2.24	2.22	2.20	1.49
**TS3-*S*$^{T50/V118}$**	2.28	2.36	2.28	2.22	2.22	2.22	1.49
**EP-*S*$^{T50/V118}$**	2.50	2.30	2.25	2.19	2.21	2.21	1.47
**d**	2.36	2.33	2.27	2.20	2.22	2.21	2.11
**σ**	0.12	0.05	0.02	0.03	0.02	0.01	0.80
**ES-*R*$^{Q59/V118}$**	3.01	2.25	2.26	2.14	2.19	2.19	4.05
**I1-*R*$^{Q59/V118}$**	3.47	2.26	2.22	2.20	2.25	2.15	3.01
**TS2-*R*$^{Q59/V118}$**	2.26	2.30	2.18	2.37	2.25	2.18	2.32
**I2-*R*$^{Q59/V118}$**	2.19	2.39	2.31	2.28	2.22	2.19	1.49
**TS3-*R*$^{Q59/V118}$**	2.29	2.37	2.31	2.27	2.21	2.19	1.49
**EP-*R*$^{Q59/V118}$**	2.86	2.24	2.28	2.18	2.20	2.17	1.48
**d**	2.68	2.30	2.26	2.24	2.22	2.18	2.31
**σ**	0.52	0.06	0.05	0.08	0.02	0.01	1.05

Table S6. Calculated B3LYP-D3 absolute energies (in a.u.). Main imaginary frequency ($\tilde{\nu}$) of transition states is reported in cm^{-1}.

Solv 1: $\varepsilon=4.0$
Solv 2: $\varepsilon=78.3$
BS1= SSD for Mn and 6-31G(d,p) for other atoms.
BS2= SSD for Mn and 6-311+G(2d,2p) for other atoms.
$E_{tot} = E_{BS2} + ZPE_{BS1} + (E_{solv,BS1} - E_{BS1})$.

	E_{BS1}	$E_{solv\ 1,BS1}$	$E_{solv\ 2,BS1}$	E_{BS2}	ZPE_{BS1}	$E_{tot,1}$	$E_{tot,2}$	$\tilde{\nu}$
**ES-$S^{Q59/V42}$**	-3710.722662	-3710.847306	-3710.909110	-3711.754308	1.611398	-3710.267553	-3710.329357	-
**TS1-$S^{Q59/V42}$**	-3710.696713	-3710.825043	-3710.892579	-3711.728352	1.608002	-3710.248680	-3710.316217	100.2*i*
**I1-$S^{Q59/V42}$**	-3710.694545	-3710.822784	-3710.890544	-3711.731109	1.610967	-3710.248382	-3710.316141	-
**TS2-$S^{Q59/V42}$**	-3710.683139	-3710.809167	-3710.875894	-3711.719555	1.611322	-3710.234261	-3710.300989	45.8*i*
**I2-$S^{Q59/V42}$**	-3710.717533	-3710.843615	-3710.908223	-3711.747671	1.614268	-3710.259484	-3710.324093	-
**TS3-$S^{Q59/V42}$**	-3710.715839	-3710.841019	-3710.904219	-3711.744862	1.609468	-3710.260574	-3710.323773	823.9*i*
**EP-$S^{Q59/V42}$**	-3710.730113	-3710.853504	-3710.914890	-3711.761160	1.615366	-3710.269185	-3710.330571	-
**ES-$R^{T50/V42}$**	-3710.724746	-3710.845400	-3710.906681	-3711.755683	1.611227	-3710.265109	-3710.326391	-
**TS1- $R^{T50/V42}$**	-3710.704730	-3710.826942	-3710.891447	-3711.735269	1.607202	-3710.250279	-3710.314784	576.6*i*
**I1-$R^{T50/V42}$**	-3710.705154	-3710.828893	-3710.894380	-3711.737071	1.610732	-3710.250077	-3710.315565	-
**TS2-$R^{T50/V42}$**	-3710.692205	-3710.818548	-3710.887662	-3711.726625	1.611961	-3710.241007	-3710.310121	125.5*i*
**I2-$R^{T50/V42}$**	-3710.724917	-3710.850539	-3710.914908	-3711.755143	1.614499	-3710.266266	-3710.330635	-
**TS3-$R^{T50/V42}$**	-3710.721818	-3710.845425	-3710.908665	-3711.750516	1.608942	-3710.265180	-3710.328421	954.2*i*
**EP-$R^{T50/V42}$**	-3710.729532	-3710.852757	-3710.915120	-3711.760775	1.615385	-3710.268616	-3710.330979	-
**ES-$S^{T50/V118}$**	-3710.722861	-3710.840188	-3710.901055	-3711.755093	1.610552	-3710.261868	-3710.322735	-
**I1-$S^{T50/V118}$**	-3710.685739	-3710.817534	-3710.885287	-3711.719939	1.613988	-3710.237746	-3710.305500	-
**TS2-$S^{T50/V118}$**	-3710.685240	-3710.816643	-3710.888858	-3711.719143	1.614225	-3710.236321	-3710.308537	77.8*i*
**I2-$S^{T50/V118}$**	-3710.734230	-3710.860477	-3710.917512	-3711.762279	1.615432	-3710.273094	-3710.330129	-
**TS3-$S^{T50/V118}$**	-3710.730567	-3710.855844	-3710.917623	-3711.756853	1.609529	-3710.272600	-3710.334380	983.8*i*
**EP-$S^{T50/V118}$**	-3710.743872	-3710.867695	-3710.928524	-3711.771261	1.616433	-3710.278650	-3710.339479	-
**ES-$R^{Q59/V118}$**	-3710.723134	-3710.843925	-3710.904944	-3711.755998	1.611418	-3710.265371	-3710.326390	-
**I1-$R^{Q59/V118}$**	-3710.693481	-3710.824151	-3710.892899	-3711.727391	1.614633	-3710.243428	-3710.312176	-
**TS2-$R^{Q59/V118}$**	-3710.678472	-3710.814900	-3710.884194	-3711.711631	1.612615	-3710.235444	-3710.304737	113.8*i*
**I2-$R^{Q59/V118}$**	-3710.728409	-3710.856794	-3710.919504	-3711.758331	1.616033	-3710.270682	-3710.333392	-
**TS3-$R^{Q59/V118}$**	-3710.722658	-3710.848889	-3710.909858	-3711.749609	1.609382	-3710.266458	-3710.327427	1025.2*i*
**EP-$R^{Q59/V118}$**	-3710.739222	-3710.868802	-3710.929246	-3711.768523	1.615957	-3710.282146	-3710.342590	-
**$TS2_{F19A}$-$S^{T50/V118}$**	-3518.926433	-3519.053145	-3519.118545	-3519.910756	1.557913	-3518.479554	-3518.540289	68.1*i*
**$TS2_{F19A}$-$S^{Q59/V42}$**	-3518.919434	-3519.050340	-3519.106161	-3519.902205	1.560763	-3518.472351	-3518.535863	105.1*i*
**$TS2_{F19A}$-$R^{T50/V42}$**	-3518.915321	-3519.039885	-3519.118545	-3519.902020	1.556997	-3518.469587	-3518.544954	110.4*i*
**$TS2_{W120A}$-$S^{T50/V118}$**	-3387.336888	-3387.463483	-3387.508958	-3388.285915	1.528164	-3386.884346	-3386.930850	182.7*i*
**TS2 $_{W120A}$-$S^{Q59/V42}$**	-3387.300621	-3387.436399	-3387.521013	-3388.250998	1.528485	-3386.858291	-3386.944266	108.5*i*
**TS2 $_{W120A}$-$R^{T50/V42}$**	-3387.334908	-3387.457649	-3387.526356	-3388.285714	1.527553	-3386.880902	-3386.947220	86.9*i*
**ES_{mut}-$S^{Q59/V42}$**	-3499.241496	-3499.364902	-3499.423745	-3500.219661	1.499634	-3498.843432	-3498.908217	-
**ES_{mut}-$R^{T50/V42}$**	-3499.244912	-3499.366406	-3499.428049	-3500.220199	1.499594	-3498.842098	-3498.903742	-
**ES_{mut}-$S^{T50/A118}$**	-3499.249688	-3499.365759	-3499.424382	-3500.226421	1.500067	-3498.842425	-3498.901048	-
**ES_{mut}-$R^{Q59/A118}$**	-3499.241019	-3499.360944	-3499.428009	-3500.219873	1.499575	-3498.840223	-3498.903024	-
**$TS2_{mut}$-$S^{T50/A118}$**	-3499.206977	-3499.334705	-3499.399724	-3500.186721	1.501191	-3498.813258	-3498.878277	47.8*i*
**$TS2_{mut}$-$R^{T50/V42}$**	-3499.208355	-3499.330441	-3499.395668	-3500.189180	1.498360	-3498.812906	-3498.878132	114.4*i*

Technical details for MD simulation

The force field parameters for **Bnz** were obtained from HF/6-31G geometry optimization of each species (using the Gaussian16 D.01 software) [1] by using the Antechamber program of the Amber Tools software package, employing the General Amber Force Field (GAFF) [2] and the Restrained Electrostatic Potential (RESP) methods [3]. The substrate was docked to *Gt*HNL crystallographic structure (pdb 4BIF) using Autodock vina software and lowest energy docked pose was later adopted for MD simulations. *Gt*HNL:Bnz model was positioned in an orthorhombic box containing TIP3P water molecules (up to 10 Å from the protein),adding counter-ions to neutralize the system. For proteins, ff14SB [4] was selected. After geometry minimization, the system was pre-equilibrated up to 300 K using langevin thermostat om NVT ensamble.

In the production phase, the NPT ensemble was considered at a constant pressure of 1 bar, using the Berendsen barostat and a time constant of τp = 2.0 ps, for 2x100 ns, with an integration step of 2 fs, employing the SHAKE algorithm and the particle mesh Ewald summation approach, as implemented in the GROMACS program. [5] With a cut-off distance of 12 Å, the electrostatic potential and long-range electrostatic interactions were computed.

References

[1] Frisch, M. J. ea; Trucks, G. W.; Schlegel, H. B.; Scuseria, G. E.; Robb, M. A.; Cheeseman, J. R.; Scalmani, G.; Barone, V.; Petersson, G. A.; Nakatsuji, H. Gaussian 16, Revision C. 01. Gaussian, Inc., Wallingford CT 2016.

[2] Wang, J.; Wolf, R. M.; Caldwell, J. W.; Kollman, P. A.; Case, D. A. Development and Testing of a General Amber Force Field. J Comput Chem 2004, 25 (9), 1157–1174.

[3] Wang, J.; Cieplak, P.; Kollman, P. A. How Well Does a Restrained Electrostatic Potential (RESP) Model Perform in Calculating Conformational Energies of Organic and Biological Molecules? J Comput Chem 2000, 21 (12), 1049–1074.

[4] Maier, J. A.; Martinez, C.; Kasavajhala, K.; Wickstrom, L.; Hauser, K. E.; Simmerling, C. Ff14SB: Improving the Accuracy of Protein Side Chain and Backbone Parameters from Ff99SB. J Chem Theory Comput 2015, 11 (8), 3696–3713.

[5] Abraham, M. J.; Murtola, T.; Schulz, R.; Páll, S.; Smith, J. C.; Hess, B.; Lindahl, E. GROMACS: High Performance Molecular Simulations through Multi-Level Parallelism from Laptops to Supercomputers. SoftwareX 2015, 1–2, 19–25.
